# Genomic and transcriptomic comparisons of closely related malaria parasites differing in virulence and sequestration pattern

**DOI:** 10.12688/wellcomeopenres.14797.2

**Published:** 2018-12-06

**Authors:** Jing-wen Lin, Adam J. Reid, Deirdre Cunningham, Ulrike Böhme, Irene Tumwine, Sara Keller-Mclaughlin, Mandy Sanders, Matthew Berriman, Jean Langhorne

**Affiliations:** 1 Malaria Immunology laboratory, Francis Crick Institute, London, NW1 1AT, UK; 2 Division of Pediatric Infectious Diseases, Sichuan University and Collaboration Innovation Centre, Chengdu, 610041, China; 3 Parasites & Microbes, Wellcome Trust Sanger Institute, Cambridge, CB10 1SA, UK

**Keywords:** Malaria, rodent, Plasmodium chabaudi, multi-gene families, virulence, parasites, evolution, host-parasite interactions, genomics, transcriptomics

## Abstract

**Background:** Malaria parasite species differ greatly in the harm they do to humans. While *P. falciparum* kills hundreds of thousands per year, *P. vivax* kills much less often and *P. malariae* is relatively benign. Strains of the rodent malaria parasite *Plasmodium chabaudi* show phenotypic variation in virulence during infections of laboratory mice. This make it an excellent species to study genes which may be responsible for this trait. By understanding the mechanisms which underlie differences in virulence we can learn how parasites adapt to their hosts and how we might prevent disease.

**Methods:** Here we present a complete reference genome sequence for a more virulent *P. chabaudi* strain, PcCB, and perform a detailed comparison with the genome of the less virulent PcAS strain.

**Results:** We found the greatest variation in the subtelomeric regions, in particular amongst the sequences of the *pir* gene family, which has been associated with virulence and establishment of chronic infection. Despite substantial variation at the sequence level, the repertoire of these genes has been largely maintained, highlighting the requirement for functional conservation as well as diversification in host-parasite interactions. However, a subset of *pir* genes, previously associated with increased virulence, were more highly expressed in PcCB, suggesting a role for this gene family in virulence differences between strains. We found that core genes involved in red blood cell invasion have been under positive selection and that the more virulent strain has a greater preference for reticulocytes, which has elsewhere been associated with increased virulence.

**Conclusions:** These results provide the basis for a mechanistic understanding of the phenotypic differences between *Plasmodium chabaudi* strains, which might ultimately be translated into a better understanding of malaria parasites affecting humans.

## Introduction

Malaria is a disease caused by parasites of the genus *Plasmodium.* While some species are highly virulent, frequently causing disease and often death, others are usually asymptomatic. Our understanding of what makes some species more virulent than others is limited, and studying this in humans is extremely difficult. Because of this, species of *Plasmodium* that infect rodents have become important models for understanding malaria in humans. Four such species ( *Plasmodium yoelii*, *P. berghei*, *P. chabaudi* and *P. vinckei*), isolated from wild thicket rats in Africa, have been adapted to grow in laboratory rodents. While these species reproduce many of the biological and pathological characteristics of human malaria parasites, *P. chabaudi* is the only species that produces a chronic blood-stage infection in laboratory mice. Many important phenotypes of *P. falciparum,* the most deadly species in humans, are mimicked by *P. chabaudi*. The latter invades both normocytes and reticulocytes, and causes anemia ( Lamb & Langhorne, 2008). Moreover, the infected red blood cells can adhere to endothelial cells in the microvasculature of host organs (sequestration) ( Brugat *et al*., 2014; Gilks *et al*., 1990). Because of these features, this model is widely used to study malarial immunology and pathology. Several strains of *P. chabaudi* have been isolated, which give rise to infections with differing parasite burdens and severity of clinical manifestations ( Cheesman *et al*., 2006; Lamb & Langhorne, 2008; Mackinnon & Read, 1999). They therefore could provide good models to determine parasite and host contributions to virulence.

It is known that the CB isolate of *P. chabaudi* (PcCB) is more virulent than the AS isolate (PcAS) in infections of mice ( Cheesman *et al*., 2006). We wanted to understand the possible genomic and transcriptomic basis for the differences in virulence between these strains. A complete genome sequence was generated for PcCB to complement the existing data for PcAS ( Brugat *et al*., 2017) and enable a detailed genomic comparison. As expected, differences were most common among genes known or expected to be involved in interaction with the host. They were particularly concentrated in the subtelomeres, where large gene families involved in host-parasite interactions such as *pir* ( *Plasmodium* interspersed repeats), evolve very rapidly. However, despite frequent rearrangements and gene conversions generating diversity at the sequence level, the repertoires of *pir* genes were almost identical. Furthermore, the patterns of AAPL (acute-associated *pir* gene locus) and ChAPL (chronicity-associated *pir* gene locus) *pir* gene loci involved in acute and chronic infections, which we identified previously ( Brugat *et al*., 2017), seem to have been largely conserved. This is the first report of a comparison of complete genomes from strains of the same non-falciparum *Plasmodium* species, enabling rearrangements in subtelomeres over a relatively short evolutionary timescale to be studied.

We have previously shown that mosquito-transmitted (MT) infections, which include the pre-erythrocytic stages, reduce the virulence of infections with both PcAS and PcCB and found evidence in PcAS that this may be related to changes in the expression of the parasite *pir* genes ( Spence *et al*., 2013). Transcriptome analysis showed that attenuation of virulence in MT PcCB compared to serially blood passaged (SBP) parasites is associated with expression of a similar repertoire of *pir* genes to that seen for PcAS. However, SBP PcCB parasites express a greater number of the L1-type *pir* genes, associated with virulence in SBP PcAS ( Spence *et al*., 2013). This suggests that *pir* genes may have a role in variation in virulence between strains, as well as between MT and SBP parasites of the same strain.

Our findings provide a basis for understanding what drives differences in virulence between parasite strains in realistic model infections. We hope that, by examining these differences more closely, a better understanding of the genomic and regulatory underpinnings of virulence in *P. chabaudi*, and more broadly *Plasmodium* parasites, will be uncovered.

## Methods

### Mice

Female C57BL/6 mice aged 6–8 weeks (body weight ranging from 15–20 g) from the SPF unit at the Francis Crick Institute Mill Hill Laboratory were used in this study. The number of mice used in each experiment is indicted below. Mice were housed under reverse light conditions (light 19.00–07.00, dark 07.00–19.00 GMT) at 20–22°C, and had continuous access to mouse breeder diet and water. This study was carried out in accordance with the UK Animals (Scientific Procedures) Act 1986 (Home Office licence 80/2538 and 70/8326), and was approved by The Francis Crick Institute Ethical Committee.

### Parasites

Cloned lines of *Plasmodium chabaudi chabaudi* AS and CB were originally obtained from David Walliker, University of Edinburgh, UK and SBP through mice by injection of infected red blood cells (iRBC) at the MRC National Institute for Medical Research, UK and cryopreserved as previously described ( Spence *et al*., 2013). *P. c. chabaudi* AS and CB lines PcASluc _230p_ and PcCBluc _230p_ expressing luciferase under the control of the constitutive promoter *eef1a* ( Lin *et al*., 2017) were used for *in vivo* imaging analysis.

### Infections and gametocyte counts

For serially blood passaged infection (SBP), infections were initiated by intraperitoneal (i.p.) injection of 10 ^5^ red blood cells infected with PcAS or PcCB. For mosquito transmitted (MT) infection, mice were infected with 20 mosquitoes infected PcAS or PcCB following a protocol described previously ( Spence *et al*., 2012). The course of infection was monitored on Giemsa-stained thin blood films by enumerating the percentage of RBC infected with asexual parasites (parasitemia). The limit of detection for patent parasitemia was 0.01% infected erythrocytes. Core body temperature was measured with an infrared surface thermometer (Fluke); body weight change of the mice was calculated relative to a baseline measurement taken before infection; and erythrocyte density was determined on a VetScan HM5 haematology system (Abaxis).

In total, seven mice per group were infected with PcAS or PcCB via mosquito transmission to monitor the course of infection, red blood cell loss, temperature and body weight changes. Groups of 5–7 mice were infected with PcAS or PcCB via MT or SBP routes to monitor gametocyte productions. The percentage of gametocytes were counted on Giemsa stained slides at day 7 or 14 post blood-stage infection.

### *In vivo* imaging

Two experiments were carried out, one with 3 mice per group and the other with 6 mice per group. C57BL/6 mice were infected with 20 mosquitoes infected with PcASluc _230p_ and PcCBluc _230p_. At days 6 and 9 post blood stage infection, the total parasite load was determined using a luciferase assay system (Promega) and bioluminescence quantitation on a Tecan Infinite plate reader from 2 μl tail blood when the parasites were at late trophozoite stage ( Brugat *et al*., 2014; Lin *et al*., 2017). The level of sequestration in different organs was investigated during schizogony. After administration of 150 mg/kg D-Luciferin (Perkin-Elmer), terminal anaesthesia (Pentobarbital 20% w/v Injected intraperitoneally to give 0.1 ml/10g of body weight) and intensive systemic perfusion, the luciferase activities in *ex vivo* organs were measured using an IVIS Lumina (Perkin-Elmer). The relative ratio of sequestration was calculated as the level of luciferase activity per organ (total flux per second) against the total parasite load measured in peripheral blood before schizogony (relative light unit, RLU).

### Reticulocyte staining

At day 7 of SBP infections, brilliant cresyl blue (BCB, sigma) and Giemsa stained thin blood films (5 mice per group) were made as previously described ( Taylor-Robinson & Phillips, 1994). The percentage of infected red blood cell that were BCB positive were enumerated by microscopy.

### DNA sequencing, assembly and annotation of PcCB genome

High-molecular-weight DNA was prepared as follows. Heparinized blood from two mice infected with *P. c. chabaudi* CB (blood-passaged parasites, day 7 post-infection) under terminal anaesthesia (as stated above) was extracted via cardiac puncture. The blood was pooled and immediately followed by DNA extraction. Leukocytes were removed by passing the infected blood through a Plasmodipur (Euro-Diagnostica) and erythrocytes were lysed by saponin (0.15% in ice-cold PBS). Infected erythrocytes were recovered by centrifugation (2,000 *g*, 10 min, 4 °C) and washed twice in ice-cold PBS. The cell pellet was resuspended in 50 mM Tris HCl pH 7.5, 50 mM EDTA pH 8.0, 100 mM NaCl, 0.5% SDS and digested with RNase A (1 mg ml ^–1^, Life Technologies) for 30 min at 37 °C. Proteinase K (Roche) was added to a final concentration of 1 mg ml ^–1^, incubated at 45 °C overnight, followed by phenol-chloroform extraction and ethanol precipitation. The extracted DNA was kept at 4 °C without any frozen step till sequencing.

For preparation of long-read sequencing libraries, 5 μg *P. c. chabaudi* CB genomic DNA was sheared to 20–25 kb by passing through a 25 mm blunt-ended needle. Single-molecule real-time (SMRT) bell template libraries were generated using the Pacific Biosciences (PacBio) issued protocol (20 kb Template Preparation Using BluePippin Size-Selection System). After 7–20 kb size-selection using the BluePippin Size-Selection System (Sage Science), the library was sequenced using P6 polymerase and chemistry version 4 (P6C4) on five SMRT cells, each with a 240 min video length.

The five SMRT cells were processed using a PacBio RSII. Reads were filtered using SMRT portal v2.2 with default parameters (minimum subread length 50, minimum polymerase read quality 75, minimum polymerase read length 50). A total of 34,079 filtered reads with an N50 of 17 Kb (~30× genome coverage) were assembled using HGAP v2.2.0 ( Stanke *et al*., 2006) with an expected coverage of 75× and other parameters as default.

The 21 assembled sequences from the HGAP assembly were BLAST-searched against the *P. chabaudi* AS v3 assembly ( Brugat *et al*., 2017), to identify nuclear chromosomes. A total of 14 unitigs represented the 14 chromosomes from the PcAS v3 assembly. All 14 had telomeric repeats at each end. For the PcAS v3 genome sequence ( Brugat *et al*., 2017), we had the benefit of a Sanger capillary sequence-based assembly (PcAS v2) to correct the indels which are an inherent problem of the PacBio long read sequencing technology. For PcCB we used 250 bp paired-end pseudoreads generated from the PcCB v1 draft genome sequence ( Otto *et al*., 2014), as we did previously for PcAS ( Brugat *et al*., 2017). These were used to correct the v2 assembly using iCORN2 v0.95 ( Otto *et al*., 2010). We transferred gene models from the PcAS v3 assembly to the corrected PcCB v2 assembly using RATT v18 ( Otto *et al*., 2011). We used Augustus v2.5.5 ( Stanke *et al*., 2006), trained on the PcAS v2 gene models with default parameters to predict a new set of gene models for the corrected PcCB v2 assembly. We kept only those that did not overlap with the RATT-transferred gene models. We then manually assessed the transferred models, annotated the new Augustus models, and renamed the locus tags.

The per-base accuracy of the assembly was improved using iCORN and pseudo-reads derived from the v1 assembly ( Otto *et al*., 2010; Otto *et al*., 2014). We annotated the PcCB genome using a combination of RATT ( Otto *et al*., 2011) and Augustus ( Stanke & Morgenstern, 2005; Stanke *et al*., 2006), to transfer genes from the AS v3 assembly ( Brugat *et al*., 2017) and to find additional genes that did not have syntenic orthologues in AS. The gene models were then manually curated and corrected where necessary.

### RNA isolation and RNA-sequencing

Female C57BL/6 mice aged between 6–8 weeks were intraperitoneally infected with 10 ^5^ iRBC of *P. c. chabaudi* AS (6 mice) or CB (6 mice) for SBP infection. For mosquito transmitted infection (MT), C57BL/6 mice were infected with 20 mosquitoes infected with AS (6 mice) or CB strain (6 mice) of *P. chabaudi*. At 7 days post blood stage infection (dpi), infected blood was collected between 11:00–11:30 GMT (reverse light cycle, see above) when more than 90% of the parasites were trophozoites. Whole blood was depleted of leukocytes by filtration (Plasmodipur, EuroProxima) and erythrocytes were lysed using saponin as described above. Purified parasite pellets were resuspended in 1 ml Tri-reagent (Ambion), snap-frozen on dry ice and kept at –80°C until use. RNA isolated with RiboPure RNA Purification Kit (Ambion) according to the manufacturer’s protocols. The quanlity and quatity of the RNA samples were verified Caliper LabChip GX (Caliper Life Sciences) and Qubit (Thermo Fisher Scientific). RNA samples were processed using Illumina Truseq and Ribo-Zero Gold kits with 15 cycles of PCR according to the manufacturer’s protocols. Sequencing libraries were then prepared using the Illumina TruSeq Stranded kit. The libraries were sequenced using a Hiseq4000, with 75 bp paired-end reads.

### *Pir* gene identification

*Pir* genes in the PcCB v2 genome were identified by BLAST-searching all inferred protein sequences against *pir* protein sequences inferred from the PcAS v3 genome ( Brugat *et al*., 2017). They were then grouped into subfamilies using hidden Markov models of rodent *pir* gene subfamilies ( Otto *et al*., 2014). We used hmmsearch from HMMer i1.1rc3 ( Eddy, 2011) with an E-value cutoff of 1e-10 and took the best hit to a subclade. We thus identified 208 *pir* genes, each assigned to a previously defined rodent malaria *pir* gene subclade.

### Orthologue identification

OrthoMCL V1.4 ( Li *et al*., 2003) was used to identify orthologues between PcAS and PcCB. An all-vs-all BLAST ( blastall v2.2.25, -p blastp, -e 0.01) was performed on the protein sequences and these were used in running OrthoMCL with default parameters. Examination of regions lacking synteny and one-to-one orthology was carried out using the Artemis Comparison Tool ( Carver *et al*., 2005).

### Imaging of subtelomere rearrangements and gene conversion

Alignments were produced using Nucmer ( Kurtz *et al*., 2004) with *--mum*. Alignments were visualised using the Artemis Comparison Tools ( Carver *et al*., 2005).

### dN/dS analysis

By excluding pseudogenes, we were able to produce sequence alignments for 4919 orthologous gene pairs. Amino acid sequences of pairwise orthologues were aligned using Muscle v3.8.31 ( Edgar, 2004) with default options. These alignments were then used to generate alignments of DNA sequences for input into codeml, part of the PAML package ( Yang, 2007). Codeml was run twice for each pair of sequences, once for a null model, with NSsites = 1 (neutral evolution) and once for the alternative mode, with NSsites = 2 (positive selection). Each run used a simple two gene tree file, runmode = 0, seqtype = 1, CodonFreq = 2, model = 0, fix_omega = 0, omega = .4. The log-likelihood ratio test was then used to calculate the significance of an omega (dN/dS) value > 1, e.g. 2 * (lnL(alt) - lnL(null)). A p-value was derived using the chi square distribution with two degrees of freedom and these p-values were corrected for multiple hypothesis testing using the Benjamini-Hochberg method. We identified 147 orthologue pairs with corrected p-value ≤ 0.01.

### Analysis of gene expression

Reads were mapped against spliced gene sequences (exons, but not untranslated regions, UTRs) from either the PcAS v3 ( Brugat *et al*., 2017) or the PcCB v2 (this work) reference genomes using Bowtie2 v2.1.0 ( Langmead & Salzberg, 2012) (-a -X 800-x). Read counts per transcript were estimated using eXpress v1.3.0 ( Roberts & Pachter, 2013), with default parameters. Genes with an effective length cutoff below 10 in any sample were removed. Summing over transcripts generated read counts per gene.

Differential expression analysis was performed using edgeR v3.8.6 ( Robinson *et al*., 2010) on genes with ≥3 counts per million. Fisher’s exact test was used with cutoffs of false discovery rate (FDR) < 0.01 and fold change ≥2. One AS replicate (SBPAS.5) was found to be an outlier by principal components analysis and was removed from further analysis. For analysis of differential expression, the functional categories of genes were identified by orthology using GeneDB ( Logan-Klumpler *et al*., 2012) from several different *P. falciparum* data sets: invasion genes ( Hu *et al*., 2010), sexual genes ( Young *et al*., 2005) and subtelomeric (by manual inspection of chromosomes). To examine functional classes enriched amongst differentially expressed genes, we used topGO (v2.20.0) for Gene Ontology analysis, with the weight01 algorithm, the Fisher statistic, node size = 5 and False Discovery Rate ≥ 0.05 ( Alexa *et al*., 2006).

### Statistical analysis

Statistical analyses of parasitological data were made using GraphPad Prism 7. Each point represents an individual biological replicate and p-values were calculated using the Mann-Whitney U-test. Details of bioinformatic statistical analyses are provided in the relevant sections.

## Results

### MT *P. chabaudi* CB is more virulent than the AS strain in C57BL/6 mice and sequesters more in the lungs

**Figure 1.**
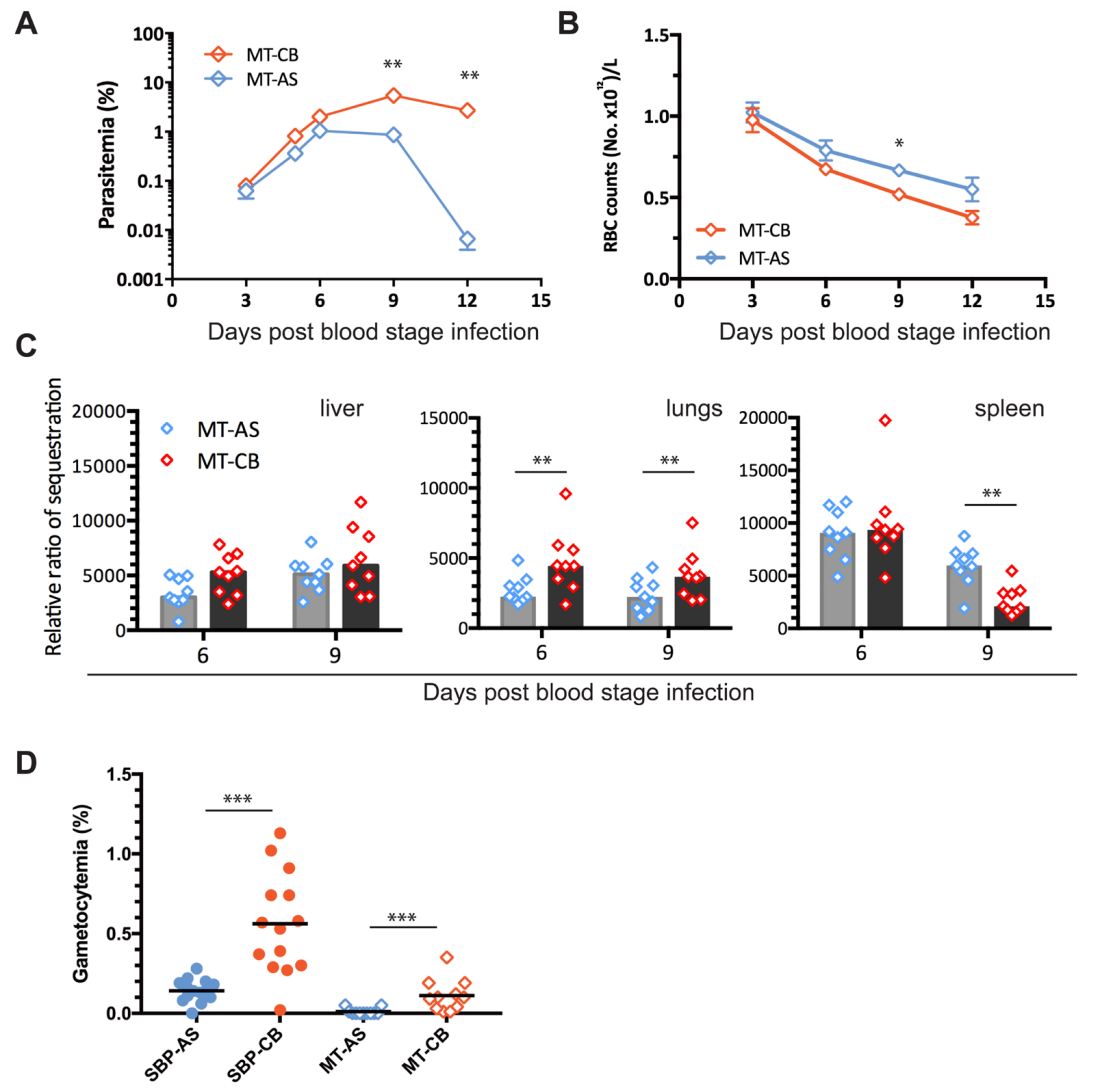
PcCB retains higher virulence than PcAS in mosquito transmitted (MT) infection in C57BL/6 mice. PcCB infection gave rise to higher parasitemia ( **A**) and lower red blood cell count ( **B**) at the peak of the acute phase of infection. Graphs show mean with SEM. Seven mice per group were used in this experiment and the data were representative of several independent experiments. ( **C**) Bar charts showing the relative ratio of sequestration in different organs, which was quantified as the level of luciferase activities in the perfused *ex vivo* organs relative to the total parasite load measured in peripheral blood at late trophozoite stage (see methods). In all bar charts, median values are shown and each dot represents an individual mouse. Mann-Whitney U-test was performed, and p values thresholds shown when significant differences were observed. Data were pooled from 2 independent experiments with 3–6 infected mice per group in each experiment. ( **D**) PcCB produced a greater proportion of gametocytes than PcAS in SBP or MT infection. The percentage of gametocyte infected RBC were counted on Giemsa stained slides at day 14 post blood-stage infection. Mann-Whitney U test was performed (***p < 0.0005). Data were pooled from 2 independent experiments with 5–7 infected mice per group in each experiment. Mann-Whitney U-test was performed (* p < 0.05, **p < 0.005, ***p < 0.0005).

Previous experiments have shown that, in infections established by SBP, PcCB is more virulent than PcAS ( Cheesman *et al*., 2006; Lamb & Langhorne, 2008; Lin *et al*., 2017) and that after mosquito transmission (MT), blood-stage parasitemia with either PcCB and PcAS is considerably lower than infections initiated with SBP parasites ( Spence *et al*., 2013). In line with these observations, we observed the PcCB strain gives rise to higher parasitemias and more red blood cell loss than the PcAS strain ( Figure 1A, B). Unlike SBP PcCB infections, there was no mortality during the acute stage of mosquito infection, and MT infections did not induce weight or body temperature loss (data not shown).

We previously observed that SBP PcAS and PcCB exhibited different sequestration levels in the lungs ( Lin *et al*., 2017) and therefore investigated whether this also happens in MT infection. We infected mice with parasites expressing luciferase constitutively throughout the life cycle (PcASluc _230p_ and PcCBluc _230p_) ( Lin *et al*., 2017). Similar to our previous finding with SBP infections, sequestration or accumulation of iRBC occurred mainly in the spleen, lungs and liver in both infections ( Brugat *et al*., 2014). While there were no significant differences in the amount of sequestration in the liver between PcAS and PcCB infections, it was significantly higher in the lungs in the PcCB infection at both 6 and 9 days post blood stage infection compared to PcAS infection ( Figure 1C), similar to our previous findings in SBP infections ( Lin *et al*., 2017). Interestingly, the relative level of sequestration/accumulation in the spleen was significantly lower in CB infection at 9 dpi ( Figure 1C).

We had previously observed that PcCB parasites transmitted more easily, producing more oocyts and sporozoites ( Spence *et al*., 2012). We looked to see whether PcCB makes an increased investment in gametocytogenesis, relative to PcAS. We found that, close to the peak of acute blood-stage infection at day 7 post blood stage infection, both PcAS and PcCB produced very few gametocytes such that it was not possible to distinguish differences between strains. However, at day 14, after the peak of parasitemia, a greater proportion of PcCB parasites were gametocytes ( Figure 1D).

Taken together, although virulence of PcCB is attenuated by mosquito-transmission similarly to PcAS, PcCB still gives rise to more severe infections with higher parasitemia, a higher level of anemia and a greater degree of sequestration in the lungs than PcAS. It also produces a greater number of gametocytes later in infection, which likely contributes to its higher transmissibility ( Spence *et al*., 2012).

### Gene content is well conserved between strains despite extensive rearrangement within the subtelomeres

To understand the genomic basis of differences in virulence between the PcAS and PcCB strains, we sequenced the genome of PcCB and compared it with the existing PcAS assembly ( Brugat *et al*., 2017). Using PacBio long-read technology, a complete assembly of the PcCB genome (version 2) was produced with every chromosome assembled telomere-to-telomere, and no gaps ( Table 1; Supplementary File 1).

**Table 1.** Genome assembly statistics *for P. chabaudi* AS and *P. chabaudi* CB genomes. Telomeric sequences were not analysed (nd) in Otto *et al.* (2014).

Variable	PcAS v3	PcCB v1	PcCB v2
Reference	Brugat *et al.*, 2017	Otto *et al.*, 2014	This work
Assembly size (Mb)	18.94	18.98	18.92
Contigs/chromosomes	14/14	460/14	14/14
N50 (Mb)	1.63	1.56	1.63
Gaps	0	349	0
Telomeric sequences/ telomeres	28/28	nd	28/28

**Figure 2.**
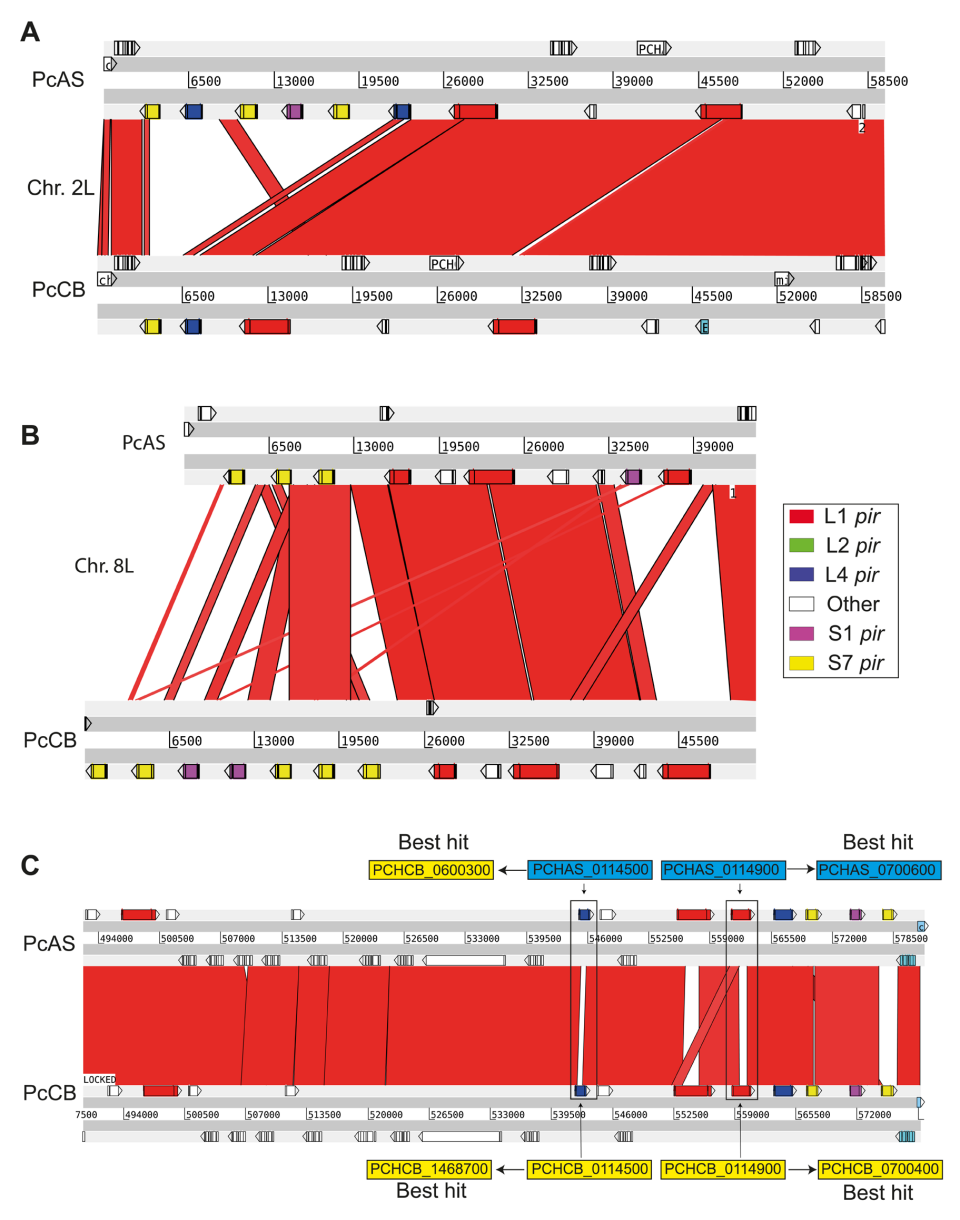
Rearrangement and gene conversion in *pir* gene loci. ( **A**) The *pir* gene locus in the subtelomere at the left-hand end of chromosome 2 (2L) contains more genes in PcAS than PcCB, suggesting an expansion in PcAS or contraction in PcCB relative to their ancestor. ( **B**) Conversely, there has been a relative expansion in PcCB at the *pir* locus on chromosome 8L. Here we use the convention of naming subtelomeres using the number (e.g. 2) and the end (L for left-hand, R for right-hand) of the chromosome. ( **C**) Shows evidence of gene conversions on chromosome 1. Orthologue pairs highlighted by black boxes appear to have undergone gene conversion. Although the gene order and *pir* gene subtypes are conserved between strains, regions within the gene, or even the whole gene, have very different sequences which match better to genes other than the orthologue. Cyan boxed indicate PcAS genes, yellow boxes indicate PcCB genes. Large arrows indicate best BLAST matches, highlighting that best matches are sometimes from different chromosomes and often the same strain, indicating gene conversion. The L1 gene PCHAS_0114900 matches better to PCHAS_0700600, in the same genome, than to the PcCB gene in the same position. Similarly the PcCB gene PCHCB_0114900 matches better to PCHCB_0700400 than to the positional gene in the other genome.

While the PcAS genome had 5177 genes, we identified 5181 in PcCB. We used orthoMCL ( Li *et al*., 2003) to identify 5008 orthologous groups of genes. From each genome, 4926 genes had one-to-one orthologues. Differences in gene content were restricted to the subtelomeric regions of chromosomes that are known to be the most variable regions across other *Plasmodium* spp. ( Lanzer *et al*., 1993). The strains had almost exactly the same number of *pir* genes, with a single extra copy in PcCB (207 in PcAS, 208 in PcCB). However, extensive gains, losses and diversification amongst *pir* genes in different orthologous clusters, has resulted in clear one-to-one orthologues being detectable for only 68 pairs of *pir* genes. Despite this, the numbers of genes in each of the *pir* subfamilies were very similar ( Table 2). Comparison of the subtelomeric genome regions containing these genes shows significant rearrangements in some places such as the left hand ends of chromosomes 2 and 8 ( Figure 2A, B). The L1-rich *pir* locus in subtelomere 4R in PcAS is essentially absent in PcCB and there has been extensive rearrangement in the ChAPL locus ( Brugat *et al*., 2017) in subtelomere 3L ( Supplementary Figure 1), although the general structure has been fairly well conserved, perhaps generating diversity rather than altering genomic structure.

**Table 2.** The numbers of *Pir* and *fam-x* gene families in *P. chabaudi* AS and CB strains. Subfamily classifications have not been performed for *pir* pseudogenes (nd).

Family	Subfamily	AS		CB	
		Complete	Pseudogene	Complete	Pseudogene
*Pir*	Ultra *pir* *	1	0	1	0
	L1	81	nd	80	nd
	L2	2	nd	3	nd
	L4	34	nd	30	nd
	S1	16	nd	17	nd
	S3	3	nd	4	nd
	S7	70	nd	73	nd
	All	207	0	208	0
*Fam-a*	-	143	2	144	1
*Fam-b*	-	26	1	30	0
*Fam-c*	-	21	1	20	1
*Fam-d*	-	17	4	17	3

*The ultra *pir*, is highly conserved in a range of Plasmodium species with large numbers of *pir* genes, e.g. *P. chabaudi*, *P. vivax* and *P knowlesi*. It is thought to be the ancestral *pir* gene and may have a very different function from other *pir* genes ( Frech & Chen, 2013).

Even when subtelomeric loci maintain exactly the same structure, variation has been introduced. Across the genome, many *pir* genes appear to be positionally conserved but do not necessarily have the most similar sequences. Those in subtelomere 1R are good examples ( Figure 2C); the genes PCHAS_0114500 and PCHCB_0114500 are syntenic and from the same L4 subtype of *pir* genes. However, their sequences differ markedly in the middle. BLAST analysis revealed that the most similar gene to PCHAS_0114500 is actually PCHCB_0600300 and the most similar gene to PCHCB_0114500 is PCHCB_1468700. Because the most similar gene is not necessarily in the other strain, but in the same strain, this suggests that there has been gene conversion. This allows genes to maintain their genomic context and *pir* gene subtype, while altering their sequence by copying from a homologous locus in the genome. Such gene conversion has been suggested to occur in the subtelomeres of *P. falciparum*, promoting divergence of the *var* gene repertoire ( Freitas-Junior *et al*., 2000). Therefore, despite similar overall numbers of *pir* genes and similar numbers of the different subtypes, there has been a great deal of diversification at the sequence level. Every subtelomere has evidence of either rearrangement of *pir* genes, or gene conversion, with gene conversion being particularly common ( Supplementary Figure 1). 

Several other subtelomeric gene families are found in rodent malaria parasites but their functions are obscure. The exception is *fam-a*, some members of which have been proposed to be involved in scavenging host phosphatidylcholine ( Fougère *et al*., 2016). A single extra *fam-a* gene was found in PcCB, three extra *fam-b* genes in PcCB, one extra *fam-c* gene in PcAS ( Table 2), one extra lysophospholipase in PcAS and two additional erythrocyte membrane antigen 1 genes in PcAS. Strain-specific duplications of exported proteins of unknown function were also found: PCHAS_0525451 in PcAS and PCHCB_0300050 in PcCB. We observed no variation in gene content amongst core genes (those outside of subtelomeres). As previously seen in comparisons between different rodent malaria species, variation in gene content is primarily limited to subtelomeric genes, particularly those of multigene families ( Otto *et al*., 2014).

### Transcriptome analysis shows a possible role for *pir* genes in virulence differences between strains

**Figure 3.**
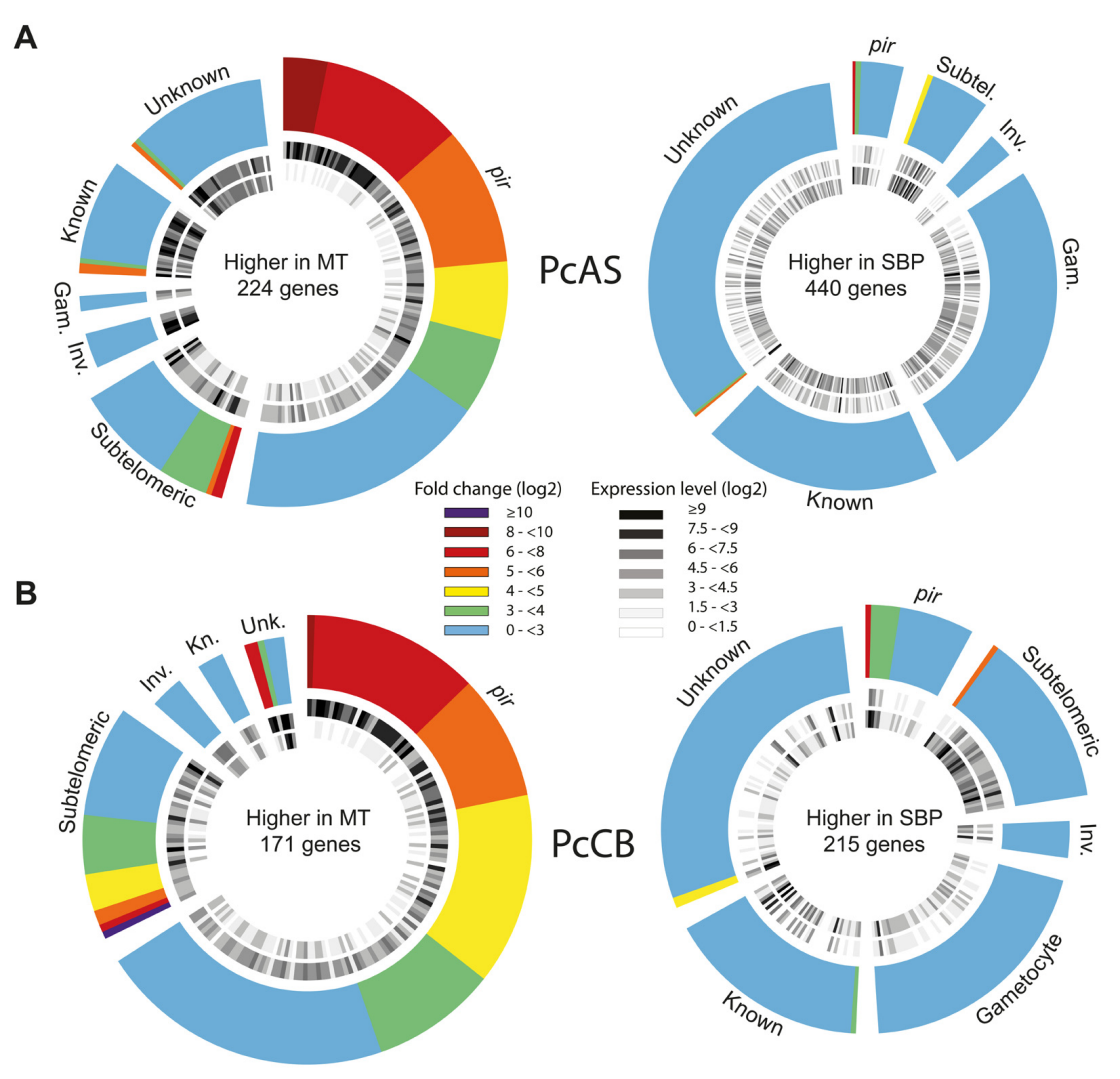
Heat maps showing differentially expressed genes between mosquito-transmitted (MT) and serially blood passaged (SBP) parasites in PcAS and PcCB. Genes more highly expressed in MT parasites than SBP parasites are shown in the left-hand hotpie, those more highly expressed in SBP than MT in the right-hand hotpie for ( **A**) PcAS and ( **B**) PcCB. Fold change is indicated on the outer ring, with warmer colours highlighting larger fold changes. The two inner tracks in black and white show the absolute expression levels in either MT or SBP samples. Genes are summarized as either *pir* genes, other subtelomeric genes, invasion-related, gametocyte-related, other known function or other unknown function.

To understand better whether *pir* genes might explain differences in virulence, we examined their expression during SBP and MT infections ( Supplementary File 2). We have shown previously that after MT infections, acute phase parasites express a much larger number of *pir* genes than SBP parasites ( Spence *et al*., 2013). We performed RNA sequencing to investigate expression differences between PcAS and PcCB in MT and SBP infections at day 7 post blood infection. We found a lower number of genes differentially expressed between SBP and MT in PcCB compared to that in PcAS: 171 genes more highly expressed in MT PcCB and 215 genes higher in SBP PcCB, compared to 224 genes higher in MT PcAS and 440 genes higher in SBP PcAS. However, we found that MT PcCB parasites had similarly increased expression of *pir* genes compared to SBP parasites as for PcAS ( Figure 3; Supplementary File 3). In line with our previous observation, the predominant *pir* gene expressed in SBP PcAS was PCHAS_1100300 ( Spence *et al*., 2013). Despite there being a syntenic orthologue for PCHAS_1100300 in PcCB, with relatively low sequence variation, this was not the predominantly expressed *pir* in SBP PcCB. A different L1 *pir* (PCHCB_1041960) was the most upregulated *pir* in SBP PcCB relative to MT. It was, however, not the most expressed, that was another L1 (PCHCB_0301170), followed by PCHCB_0101200 (a highly expressed *pir* gene, widely conserved in the genus ( Frech & Chen, 2013) and PCHCB_0301160 (L1). Therefore, SBP PcCB express several L1 *pir* genes, whereas SBP PcAS express only a single one (excepting the ultra-conserved *pir*). Given their association with virulence ( Brugat *et al*., 2017), the expression of multiple L1s in SBP PcCB is a possible cause of its increased virulence. This is unlikely to be affected by variation in gametocyte development as single-cell RNA-seq suggests that it is S-type *pir* genes that are expressed in gametocytes ( Reid *et al*., 2018).

**Figure 4.**
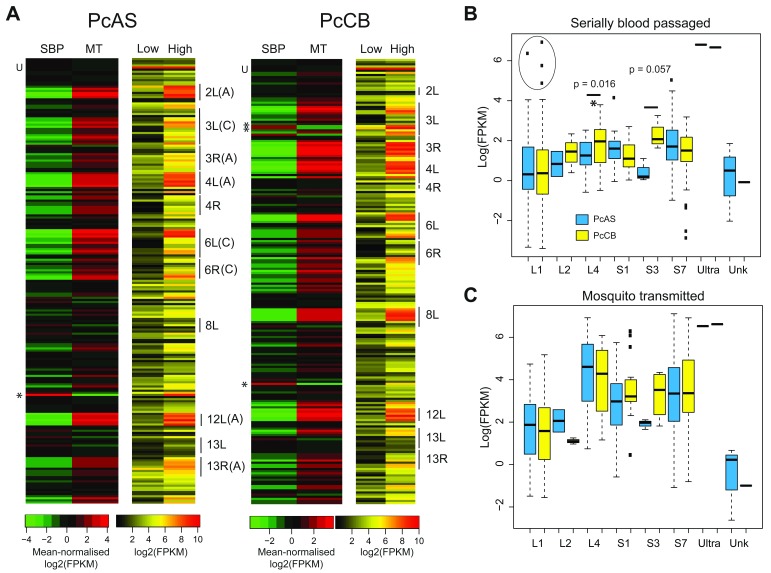
Heat maps of *pir* gene expression in PcAS and PcCB. ( **A**) Red-green heat maps show the relative expression levels of *pir* genes between serially blood passaged (SBP) and mosquito-transmitted (MT) parasites of PcAS and PcCB. Red colours indicate higher expression. Black-yellow-red heatmaps indicate the minimum and maximum expression levels of each gene, indicating which are most highly expressed (red colour) and which are in some instances expressed at very low levels, if at all (black). Genes are ordered by position in the genome from chromosome 1 to 14. Particular loci of interest e.g. 2L (left-hand end of chromosome 2) are indicated. ChAPL ‘C’ and AAPL ‘A’ loci in PcAS are also indicated. ‘U’ indicates the ultra-conserved pir, asterisk indicates L1 *pir* genes highly expressed in SBP. ( **B**, **C**) Differences in expression levels between *pir* subgroups in each strain for SBP and MT infections. Only L4 *pir* genes were significantly different in SBP parasites, having overall higher expression levels in PcCB than PcAS. S3 pir genes were nearly significant, having a large difference, but a low number of genes. Despite strong outliers, which are clearly differentially expressed (highlighted by the ring), L1s on the whole were not different between strains. There were no significant differences in expression between PcAS and PcCB *pir* subfamilies in MT parasites. P-values were calculated using two-sided Kolmogorov-Smirnov tests.

Relative to SBP parasites, 132 and 124 *pir* genes increased in expression in MT PcAS and PcCB respectively. These genes represent a range of subfamilies with similar distribution in the two strains ( Table 3). Figure 4A shows that the most differentially and highly expressed *pir* genes cluster in similar regions in PcAS and PcCB genomes. This aligns with our finding that *pir* gene subfamily repertoires are very similar between the two strains. There were, however, some specific differences. We noted above that there were *pir* gene expansions in subtelomere 2L in PcAS and 8L in PcCB. We now find that 2L is highly expressed in PcAS and 8L in PcCB, but not vice versa ( Figure 4A). 2L was identified as an AAPL, associated with acute rather than chronic infection in our earlier work ( Brugat *et al*., 2017). Here it seems that 8L in PcCB, which is similarly rich in S7 *pir* genes, may be an alternative AAPL in PcCB. *Pir* genes in subtelomere 3R are expressed more in MT PcCB than PcAS despite no change in structure and little change in sequence ( Figure 4A; Supplementary Figure 1). Despite expansion in the 6R locus in CB, the chronicity-associated ChAPL loci of AS (3L, 6L and 6R) seem to have similar expression patterns between strains ( Figure 4A). The exception is that 3L in PcCB contains several L1s highly expressed in SBP parasites. The only significant difference in expression between *pir* gene subfamilies in each strain was for L4 *pir* genes in SBP parasites ( Figure 4B,C). These were more expressed in PcCB. This subfamily of *pir* genes is found in both AAPLs and ChAPLs and is not associated with increased or decreased virulence.

**Table 3.** *Pir* subfamilies differentially expressed between mosquito-transmitted (MT) and serially blood passaged (SBP) parasites.

	Higher in MT		Higher in SBP	
Subfamily	AS	CB	AS	CB
L1	40	36	12	14
L2	2	0	0	1
L4	30	23	0	1
S1	12	14	0	0
S3	3	2	0	0
S7	45	49	5	3

The complete lists of genes differentially expressed between SBP and MT infections in each strain are shown in Supplementary File 3, along with GO term enrichments highlighting functional terms enriched amongst these genes. They suggest that there is a difference in gametocyte commitment between strains, for which the GO term ‘microtubule-based movement’ is a good indicator. In both SBP and MT infections, PcAS had a significant enrichment for microtubule-based movement (topGO Fisher statistic; p = 0.0188 and p = 4.40E-11, respectively) and higher expression of well-known gametocyte gene families such as LCCL and CPW-WPC. We do not believe that these differences affect our conclusions on *pir* genes, as gametocytes (in *P. berghei*) are known to express principally S-type *pir* genes ( Reid *et al.*, 2018) and the differences in gametocyte genes all have small changes, reflecting a low abundance of immature gametocyte forms.

### Genes involved in sexual development and red blood cell invasion are under diversifying selection

Our analysis of orthology showed us how genomes vary in terms of presence and absence of genes. It cannot tell us anything about the majority of genes, which are conserved between isolates but may have significant variation in their sequences. To determine whether shared genes have been under diversifying selection pressure in one lineage and might be associated with phenotypic differences between isolates, we looked at dN/dS between pairwise orthologues using a pairwise site model ( Yang, 2007). Genes from multigene families were excluded as orthologue identification is problematic in these cases. We identified 95 one-to-one orthologue pairs, which have likely been under diversifying selection since the PcAS and PcCB genomes diverged ( Supplementary File 4A). Gene Ontology analysis identified ‘ribonucleotide binding’ as a function enriched amongst these genes (FDR = 0.044; Supplementary File 4B). A total of seven RNA-binding genes were found to be under diversifying selection. One of these, *puf1*, is known to be involved in sequestering mRNA transcripts in female gametocytes, readying them for rapid development after fertilisation ( Shrestha *et al*., 2016). The *P. falciparum* orthologues of all seven genes showed highest expression in stage V gametocytes or ookinetes ( Lopez-Barragan *et al*., 2011) ( Supplementary Figure 2). This suggests that aspects of sexual development are under positive selection in this lineage.

**Figure 5.**
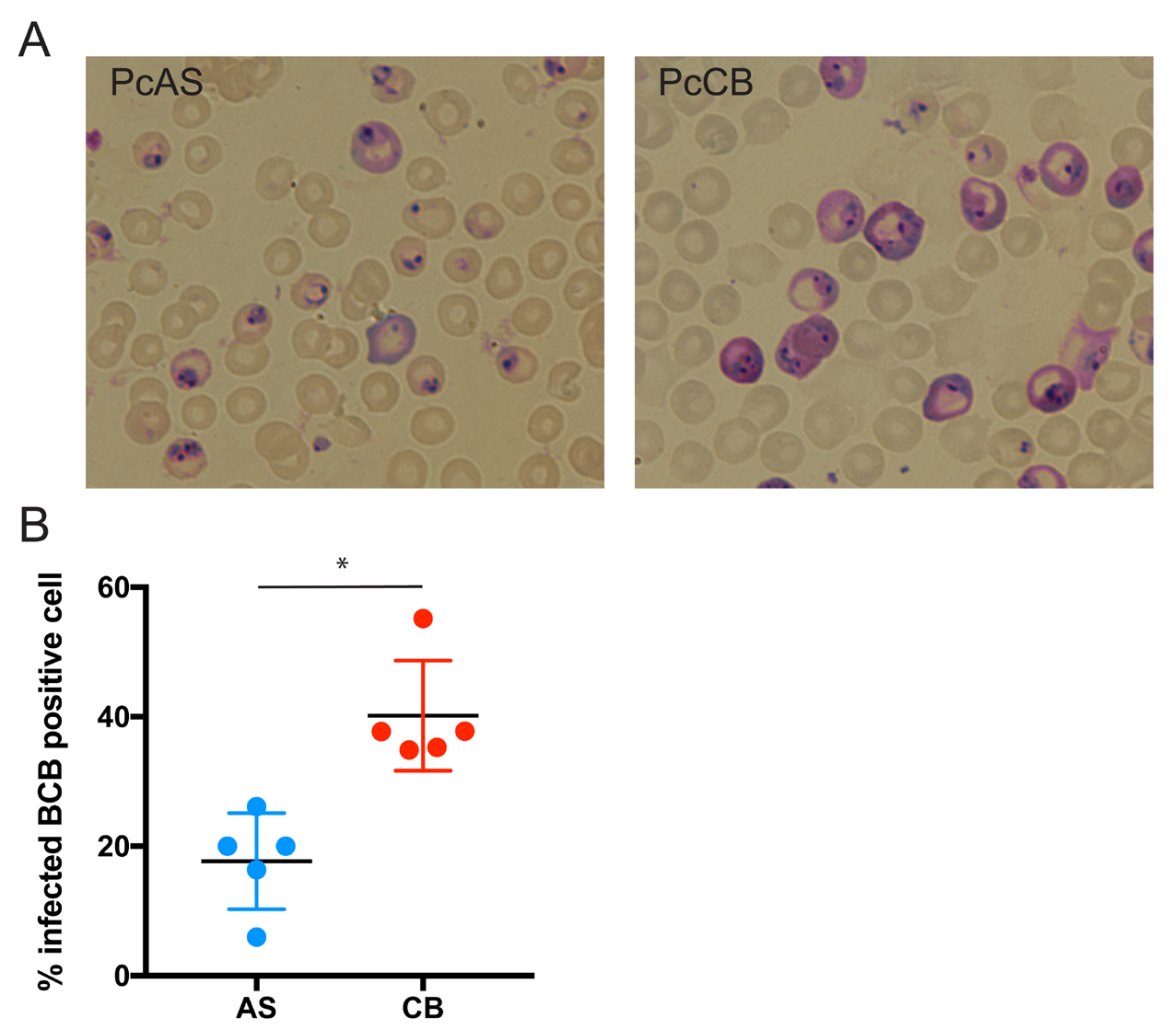
PcCB shows stronger ability to invade reticulocytes compared to PcAS. ( **A**) Microscope images showing Giemsa stained blood smear of SBP PcAS and PcCB at day 7 post infection. ( **B**) Graph showing percentages of infected red blood cell that were in reticulocytes at day 7 post SBP infections. Each dot represented one individual mouse, the lines show mean with SD. Reticulocytes were determined by brilliant cresyl blue (BCB) staining on thin blood smears. Data were representative of 2 independent experiments with 5–6 infected mice per group. Mann-Whitney U-test was performed (* p < 0.05).

Genes under diversifying selection were also enriched for the terms *pathogenesis* (FDR = 0.0044) and *host cell* (FDR = 0.006). This pointed towards genes involved in host-parasite interactions ( *maebl*, *msp1,* exported proteins; Supplementary File 4B). Among the list of diversified genes, we identified 17 that encode proteins that are potentially involved in host-parasite interactions, based on the functions of their homologues in other *Plasmodium* species ( Baldwin *et al*., 2015; Orito *et al*., 2013; Rathore *et al*., 2003; Triglia *et al*., 2009) and similar signals of positive selection in other *Plasmodium* lineages ( Forni *et al*., 2015; Sawai *et al*., 2010). Among these, six genes are associated with red blood cell recognition and invasion. Differences in preference for young (reticulocytes) versus old (normocytes) red blood cells has been linked previously with virulence. Therefore, we investigated whether PcAS and PcCB have different red cell preferences. We found that at 7 days post SBP infection, approximately 40% of PcCB-infected red blood cells were reticulocytes, while only 20% of PcAS-infected red blood cells were reticulocytes ( Figure 5). It has been proposed that parasites that are better able to invade both young and old red cells have a growth advantage and are therefore able to reach higher parasitemias, causing a higher level of anemia ( Antia *et al*., 2008).

Raw data associated with each Figure are available on GitHub and Zenodo ( Reid, 2018).

## Discussion

The rodent malaria parasite *Plasmodium chabaudi* is a good model for understanding the interactions between the parasite and its host. Previous work has shown that the PcAS and PcCB strains of this species display differences in virulence in C57BL/6 mice in both serially-blood passaged and more natural MT infections ( Cheesman *et al*., 2006; Lamb & Langhorne, 2008; Lin *et al*., 2017). Here we have explored parasitological, genomic and transcriptomic factors that might underlie this difference in virulence.

To provide a template for understanding the genomic basis of these strain differences, we have generated a complete reference genome sequence for PcCB and compared it in detail with that we previously produced for PcAS ( Brugat *et al*., 2017). As expected, we found the greatest variation in the subtelomeric regions, in particular amongst the *pir* genes. Every subtelomere has been subject to gene conversion, if not more extensive rearrangements, and these strains may already be too divergent in the subtelomeres to deconvolute the precise series of events resulting in this divergence. However, despite variation in sequence due to rearrangements and gene conversions, the general character of the different *pir* loci and in particular the frequencies of *pir* gene subtypes (e.g. L1, L2, S1, S7 etc.) were well conserved, suggesting that their proposed role in establishing chronic infection is conserved. We have previously shown that PcAS parasites transmitted between mice by serial blood passage express a narrow range of *pirs*, dominated by an L1 from outside of a ChAPL locus ( Brugat *et al*., 2017; Spence *et al*., 2013). When transmitted to a mouse via mosquito bites, these parasites begin to express a wide range of both S and L type *pir* genes, predominantly from the AAPL and ChAPL loci ( Brugat *et al*., 2017). When we compared the transcriptomes of the two strains after SBP and MT infections we observed a similar pattern in PcCB, with non-ChAPL L1 *pir* genes dominating SBP infections and AAPl and ChAPL loci dominating the MT infections. In this evolutionary timescale we see a great deal of structural and likely functional conservation of *pir* gene loci, despite extensive changes at the sequence level. However, rather than the single prominent L1 *pir* gene expressed in SBP PcAS parasites, PcCB expressed several L1 *pir* genes at high levels. Given the association between L1 *pirs* and virulence this could be further explored as a possible cause of increased virulence in SBP PcCB ( Brugat *et al*., 2017; Spence *et al*., 2013). Clues as to whether *pir* genes might affect virulence during MT are harder to identify as *pir* gene expression is much more diverse.

It has long been hypothesized that virulence is positively associated with parasite transmission rate. It has been suggested that increased virulence results in higher numbers of parasites, higher numbers of transmission stages and increased lifetime infectivity ( Mackinnon & Read, 2004). Here we showed that PcCB produces more gametocytes than PcAS after the peak of acute infection, and higher gametocyte production is likely the reason for the increased transmissibility of PcCB, evident from higher oocyst and sporozoite production ( Spence *et al*., 2012). We also found several genes involved in gametocyte development have been under diversifying selection. This further highlights the importance of the transmission stages in parasite evolution.

Virulence determinants have also been studied in the rodent malaria model P. *yoelii*. In this species, several genes involved in erythrocyte invasion have been linked with virulence, such as *Py235* ( Bapat *et al*., 2011; Pattaradilokrat *et al*., 2009), *PyEBL* ( Abkallo *et al*., 2017; Otsuki *et al*., 2009), and the HECT-like E3 ubiquitin ligase *Pyheul* ( Nair *et al*., 2017). Orthologues of *PyEBL* (PCHAS_1337300) and *Pyheul* were not under positive selection or differentially expressed between PcAS and PcCB strains. However, one *Py235* homologue, reticulocyte binding protein (PCHAS_0101100), did seem to be under diversifying selection; and two additional homologues, PCHCB_0525200 and PCHCB_0900051 were more highly expressed in PcCB than PcAS during SBP infection. We also found several other genes involved in red blood cell invasion were under diversifying selection. We demonstrated that PcCB has a greater ability to invade reticulocytes compared to PcAS, whilst retaining the ability to invade normocytes. This is in line with the previous finding that more virulent *P. chabaudi* clones were those estimated to be able to invade a greater range of RBCs ( Antia *et al*., 2008); this higher reticulocyte invasion ability may be one of the reasons why PcCB reaches higher parasitemia and causes more anemia than PcAS. It would be interesting to examine whether this is linked with differential lung sequestration and pathology. Interestingly, the more normocyte-restricted PcAS caused less lung pathology than PcCB ( Lin *et al*., 2017). It is possible that parasite strains that invades a broader range of RBC early in infection also causes more severe lung pathology compared to either reticulocyte or normocyte restricted strains. 

## Data availability

The Pacific Biosciences RSII genomic sequencing reads, used to generate the *P. c. chabaudi* CB v2 genome sequence are available from the ENA, accession number ERX662634: https://www.identifiers.org/ena.embl/ERX662634.

The assembled genome sequence and annotation can be accessed from GeneDB ( ftp://ftp.sanger.ac.uk/pub/project/pathogens/Plasmodium/chabaudi/CB_v2/).

The RNA-seq data sets used in this study have been submitted to the ENA, secondary accession number ERP110375: https://www.ebi.ac.uk/ena/data/view/PRJEB28199. These datasets are described further in Supplementary File 1.

Read counts and normalised RNA-seq data, and raw parasitological data can be accessed from GitHub ( https://github.com/adamjamesreid/Plasmodium-chabaudi-CB-genome-paper). Archived data at time of publication are available on Zenodo, DOI: https://dx.doi.org/10.5281/zenodo.1470249 ( Reid, 2018).

## Supplementary material

**Supplementary Figure 1. Alignments of subtelomeres for chromosomes 1–7 between PcAC and PcCB, showing rearrangements and gene conversions.**

Alignments were performed using nucmer and identify regions of high similarity. Matches in blue represent inversions. L, left hand end of chromosome; R, right hand end. PcAS is the upper genome in each plot, PcCB the lower one.

**Supplementary Figure 2. The P. falciparum orthologues of 7 RNA-binding proteins all showed highest expression at gametocyte V or ookinete stage.**

Colour scale shows Z score of FPKM normalised read counts of each gene ( Lopez-Barragan *et al*., 2011). Red indicates higher expression and blue indicates lower expression, and yellow indicates median expression.

**Supplementary File 1. Sequence datasets used in this work.**

Descriptions of long-read genome sequence dataset for P. chabaudi CB and short-read RNA-seq datasets for PcAS and PcCB.

**Supplementary File 2. Read counts/fragments per kilobase of transcripts per million mapped reads (FPKMs) for each RNA-seq experiment.**

(A) RNA-seq read counts for PcAS, (B) FPKMs for PcAS, (C) read counts for PcCB, (D) FPKMs for PcCB, (E) read counts for combined orthologues of PcAS and PcCB.

**Supplementary File 3. Genes differentially expressed between mosquito transmitted and blood transmitted parasites of PcAS and PcCB.**

(A) Genes differentially expressed between serially blood-passaged (SBP) PcAS and mosquito-transmitted (MT) PcAS with FDR ≤ 0.01 and fold change > 2. Direction = ‘up’ in MT, ‘down’ in MT, FDR = False Discovery Rate, logCPM = logged Counts Per Million, logFC = logged Fold Change, Desc = gene product description. (B) Genes differentially expressed between SBP PcCB and MT PcCB. Direction = ‘up’ in MT, ‘down’ in MT. (C) Genes differentially expressed between SBP PcAS and SBP PcCB. Direction = ‘up’ in PcCB, ‘down’ in PcCB. (D) Gene Ontology terms enriched amongst genes differentially expressed between SBP PcAS vs. SBP PcCB. ‘UP’ = enriched in PcCB, ‘DOWN’ = enriched in PcAS. BP = Biological Process, MF = Molecular Function, CC = Cellular Component. Frequency is the total number of occurrences of a term in the genome annotation, observed is the number of occurrences of that term in the differentially expressed genes, expected is the number of times that term ought to occur by chance, FDR is the False Discovery Rate. (E) Genes differentially expressed between MT PcAS and MT PcCB. Direction = ‘up’ in PcCB, ‘down’ in PcCB. (F) GO terms enriched amongst genes differentially expressed between MT PcAS and MT PcCB. ‘UP’ = enriched in PcCB, ‘DOWN’ = enriched in PcAS.

**Supplementary File 4. Orthologue pairs with dN/dS > 1.**

(A) Genes identified as having dN/dS > 1. Those with a role in host-parasite interactions or RNA-binding are highlighted. (B) Gene Ontology terms enriched amongst the gene list in (A).
