# Supplementary figures and images for: Genomic and transcriptomic comparisons of closely related malaria parasites differing in virulence and sequestration pattern
Source: Wellcome Open Res. 2018 Dec 6;3:142. Originally published 2018 Nov 2. [Version 2] doi: 10.12688/wellcomeopenres.14797.2 (PMC6259598; doi:10.12688/wellcomeopenres.14797.2)

1L

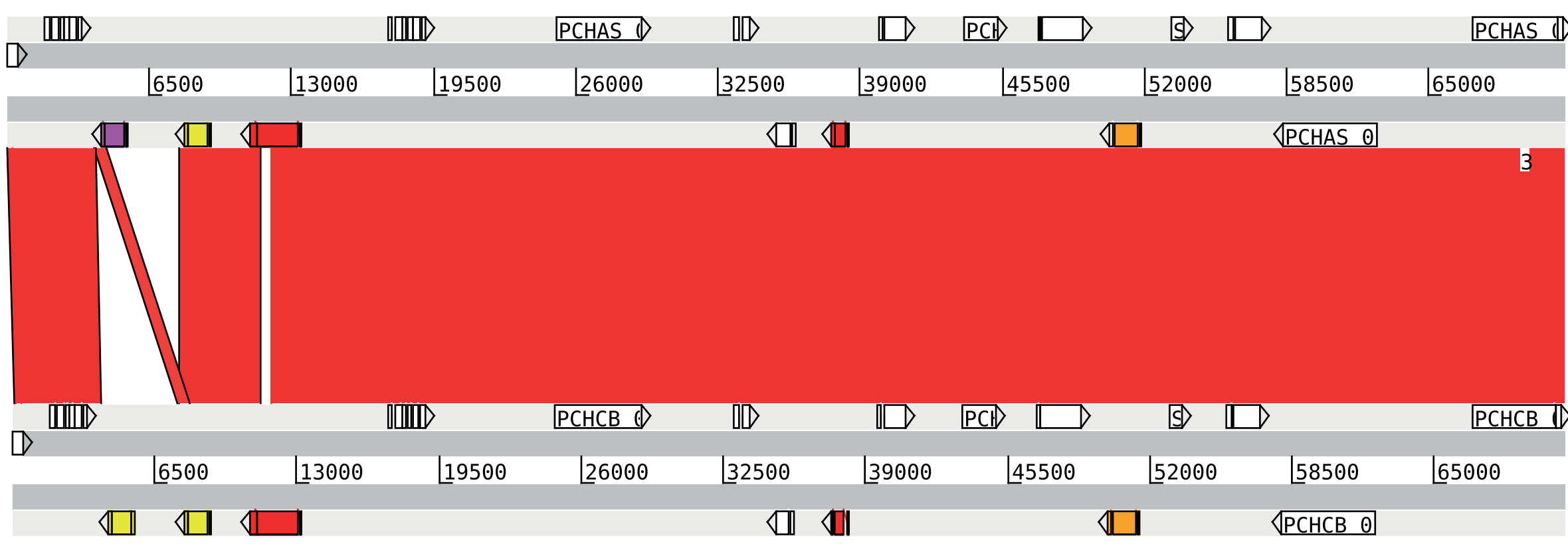

2L

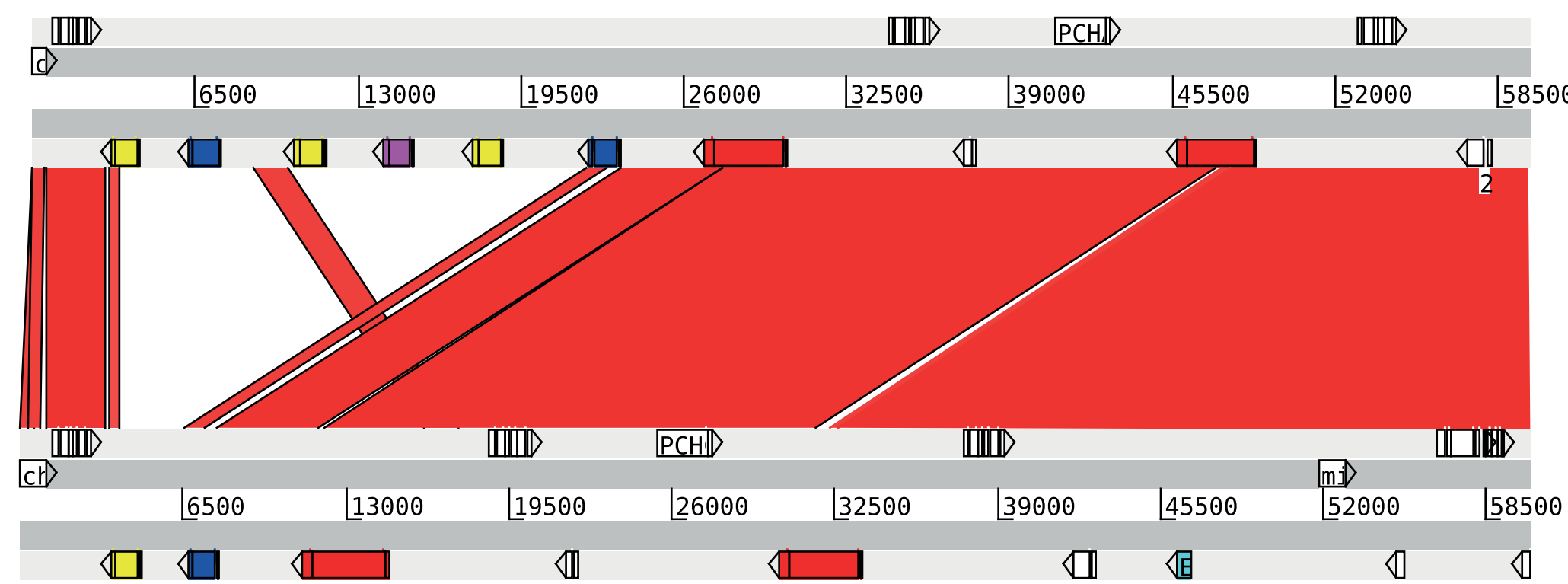

3L

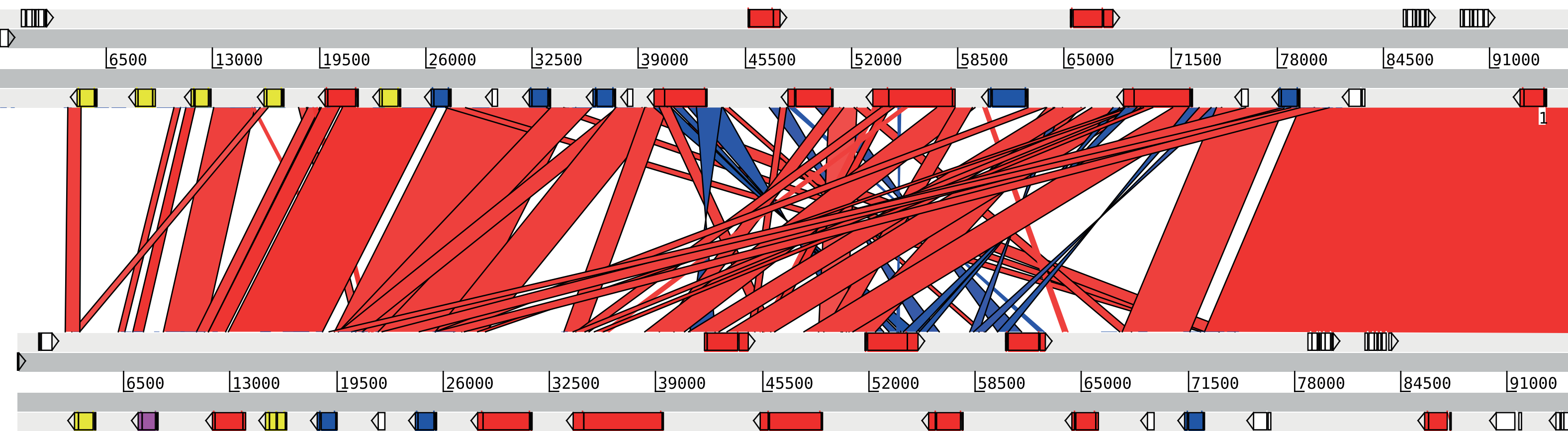

4L

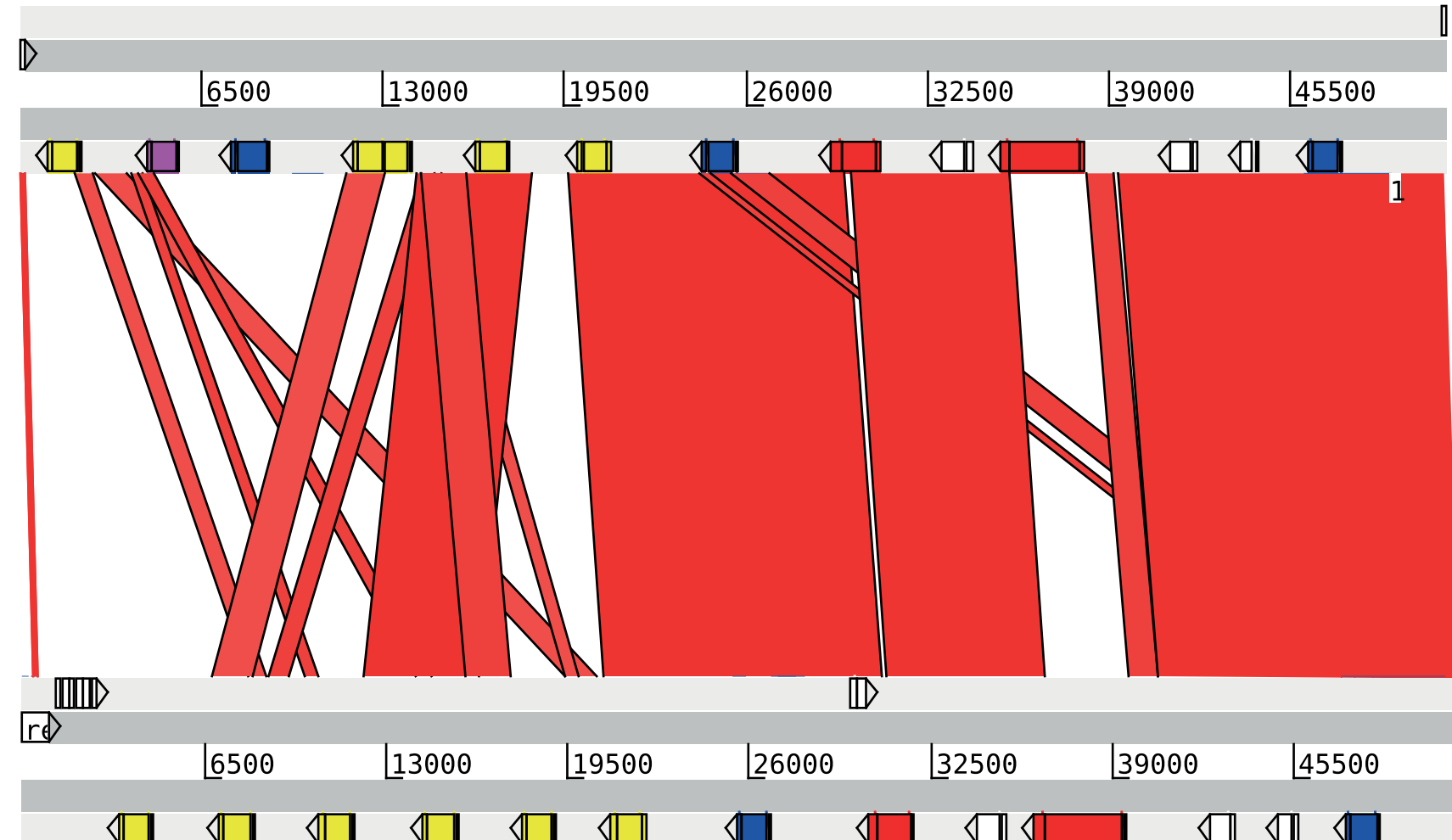

5L

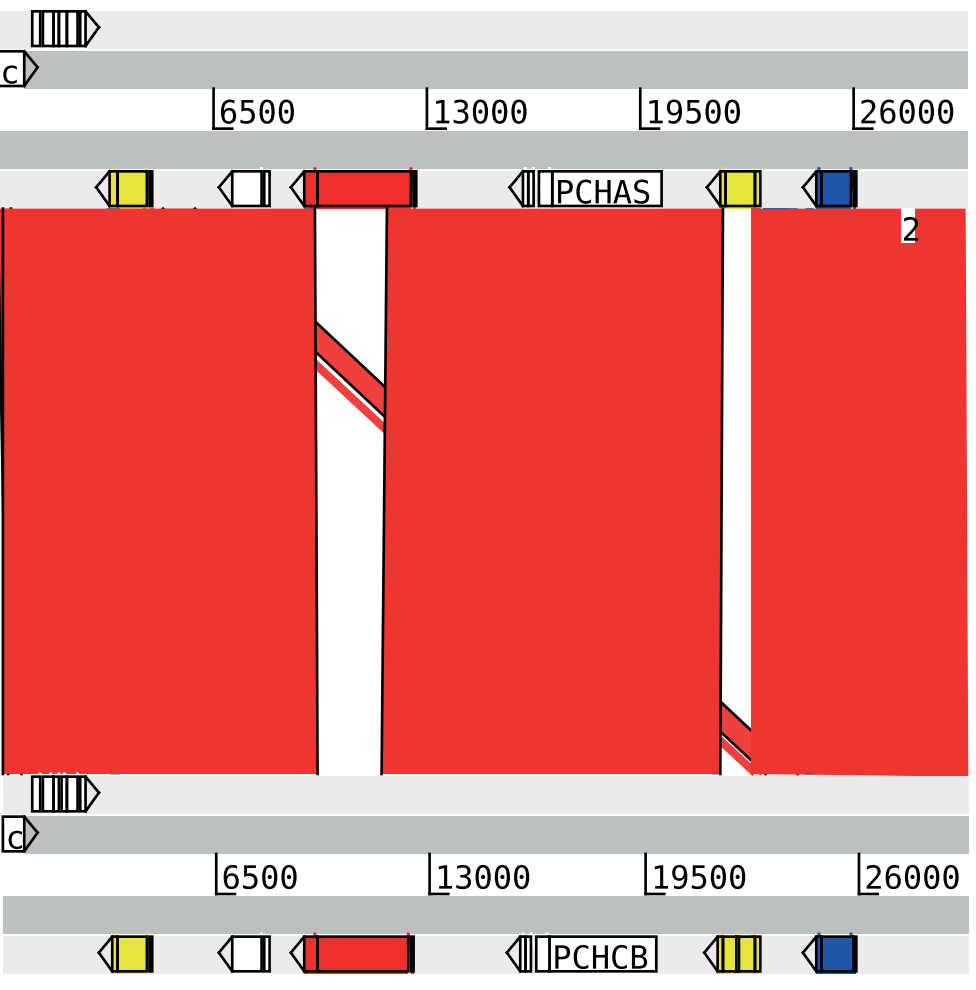

6L

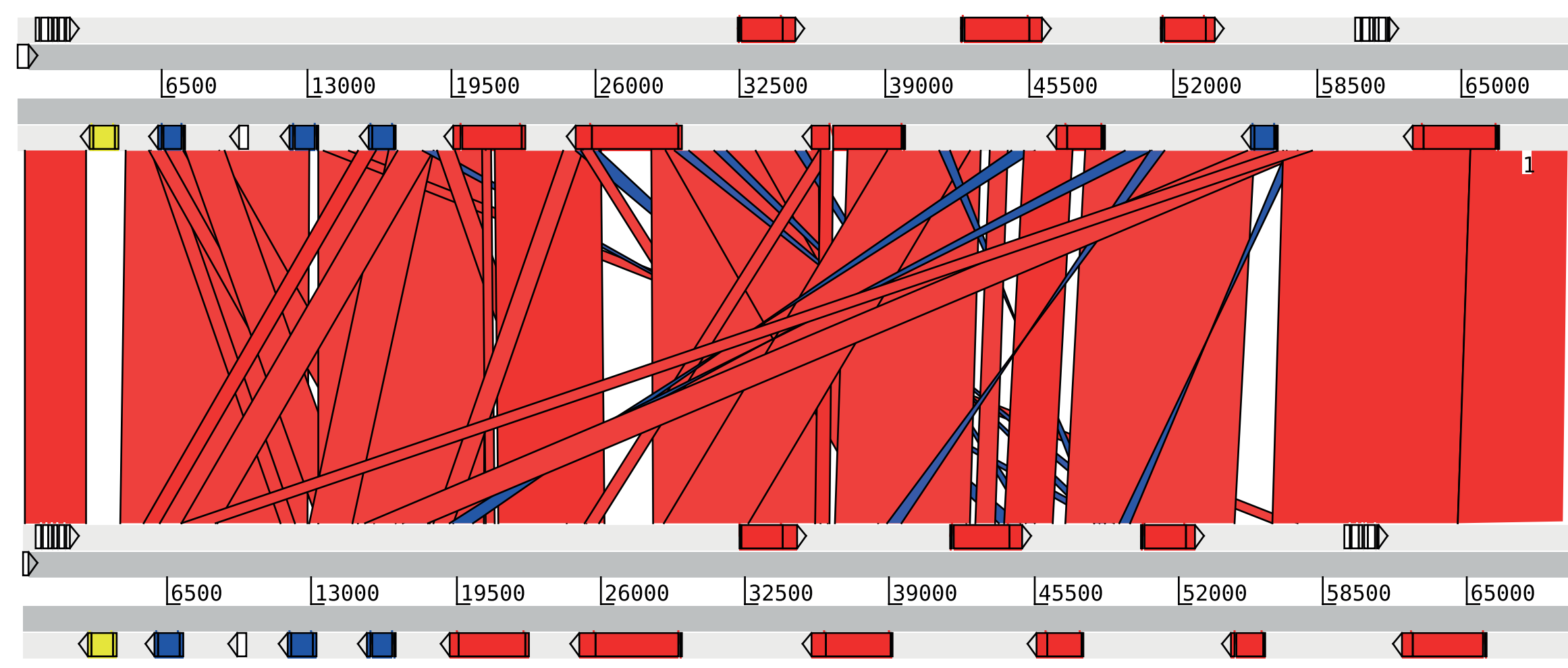

7L

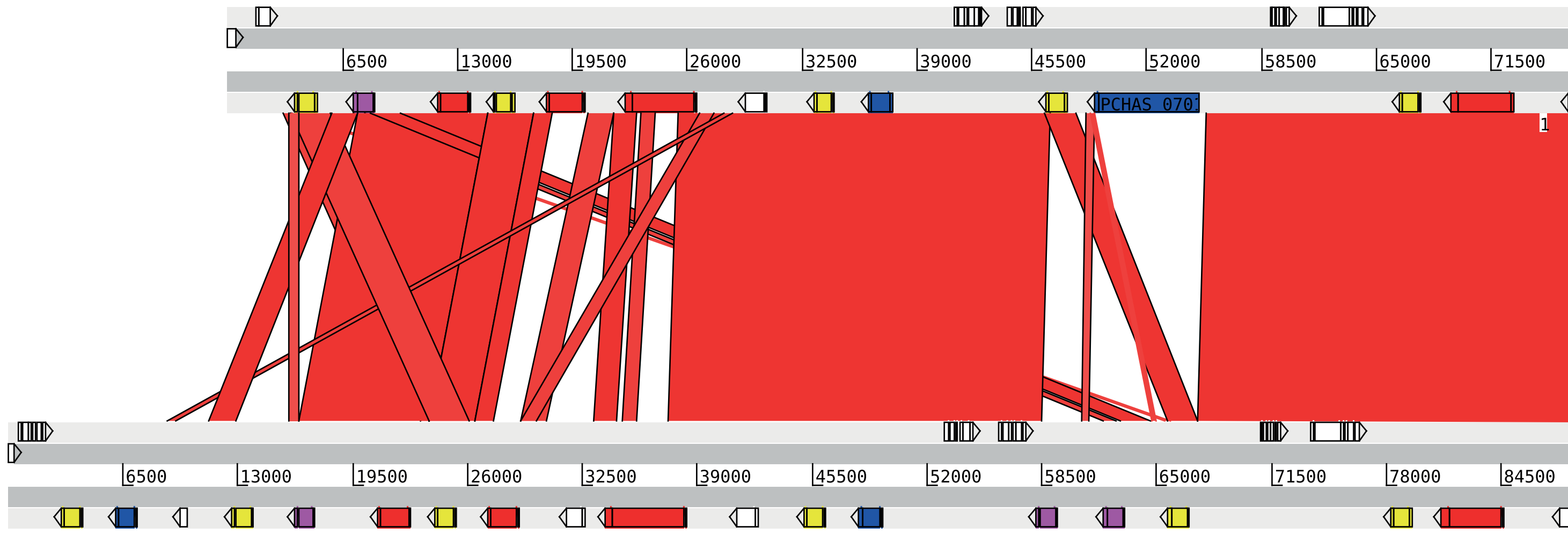

8L

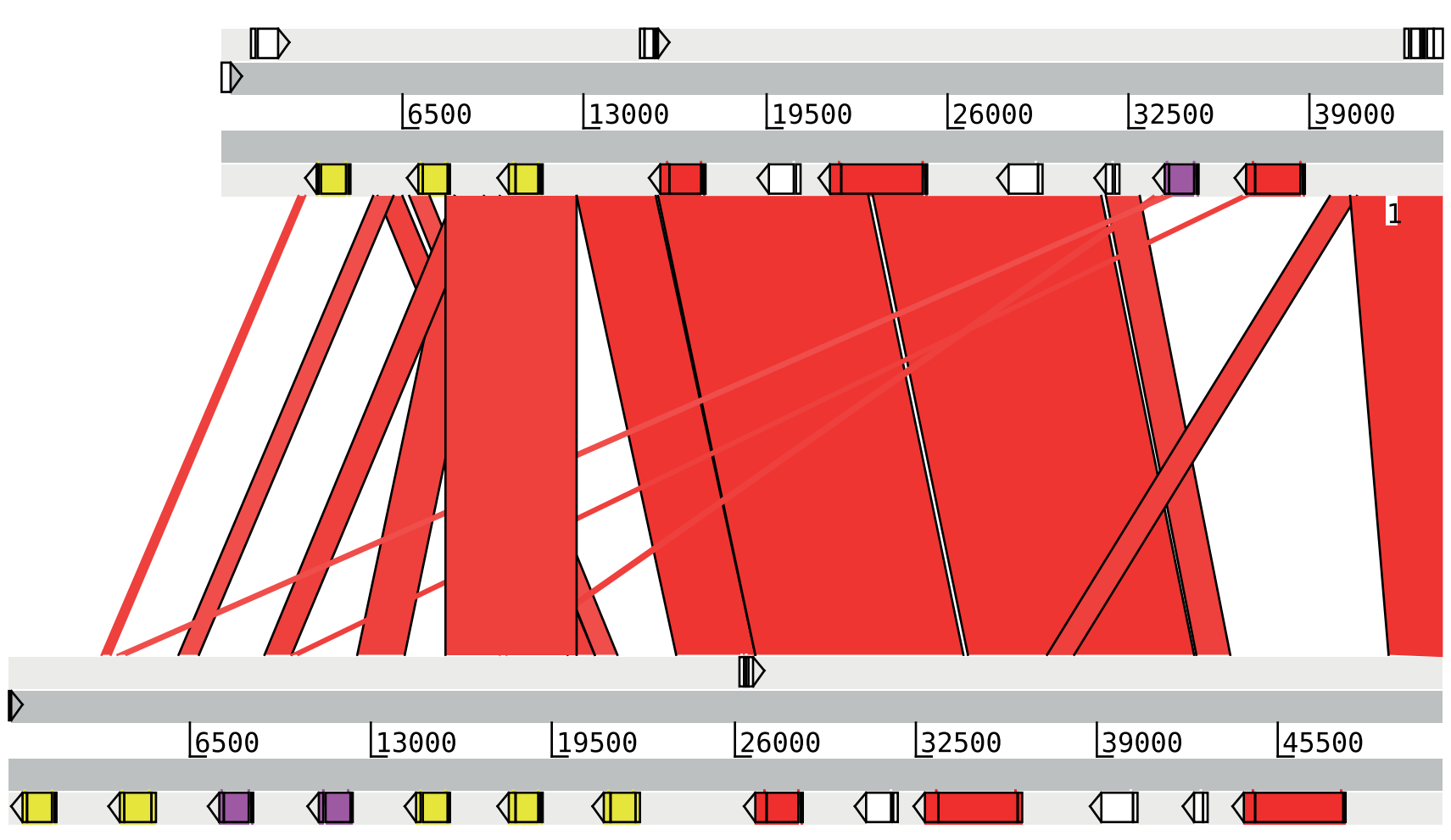

9L

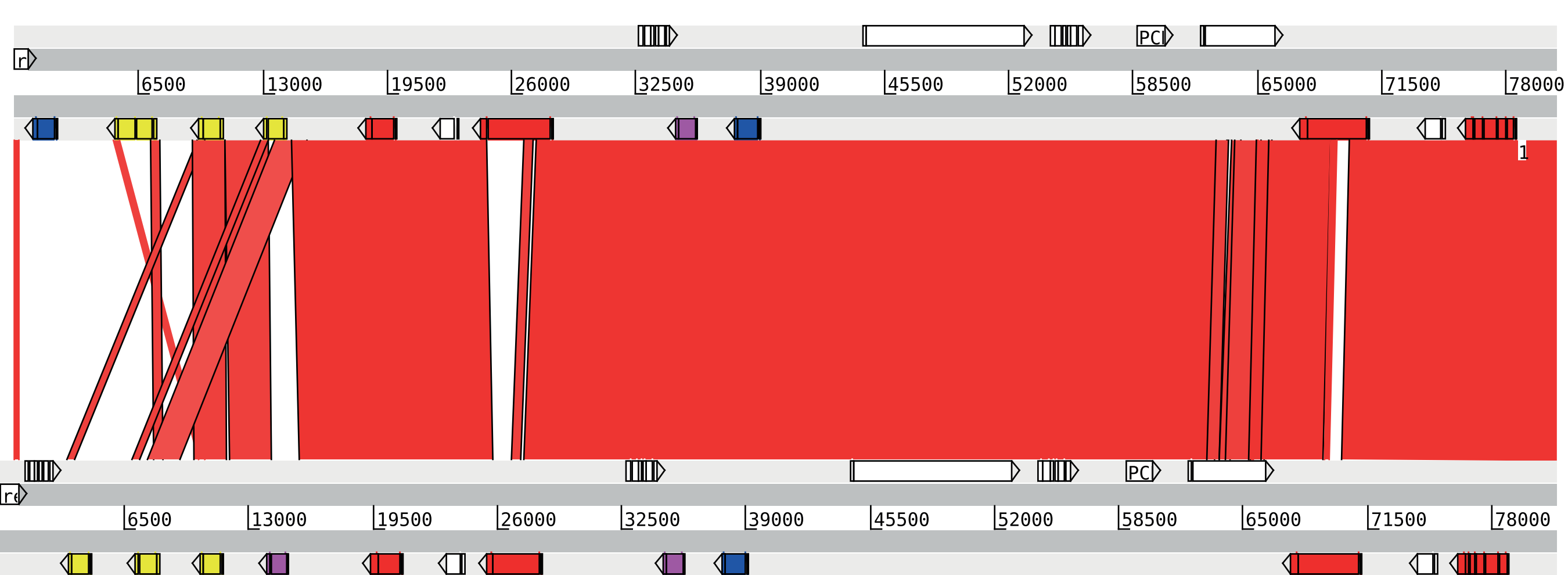

10L

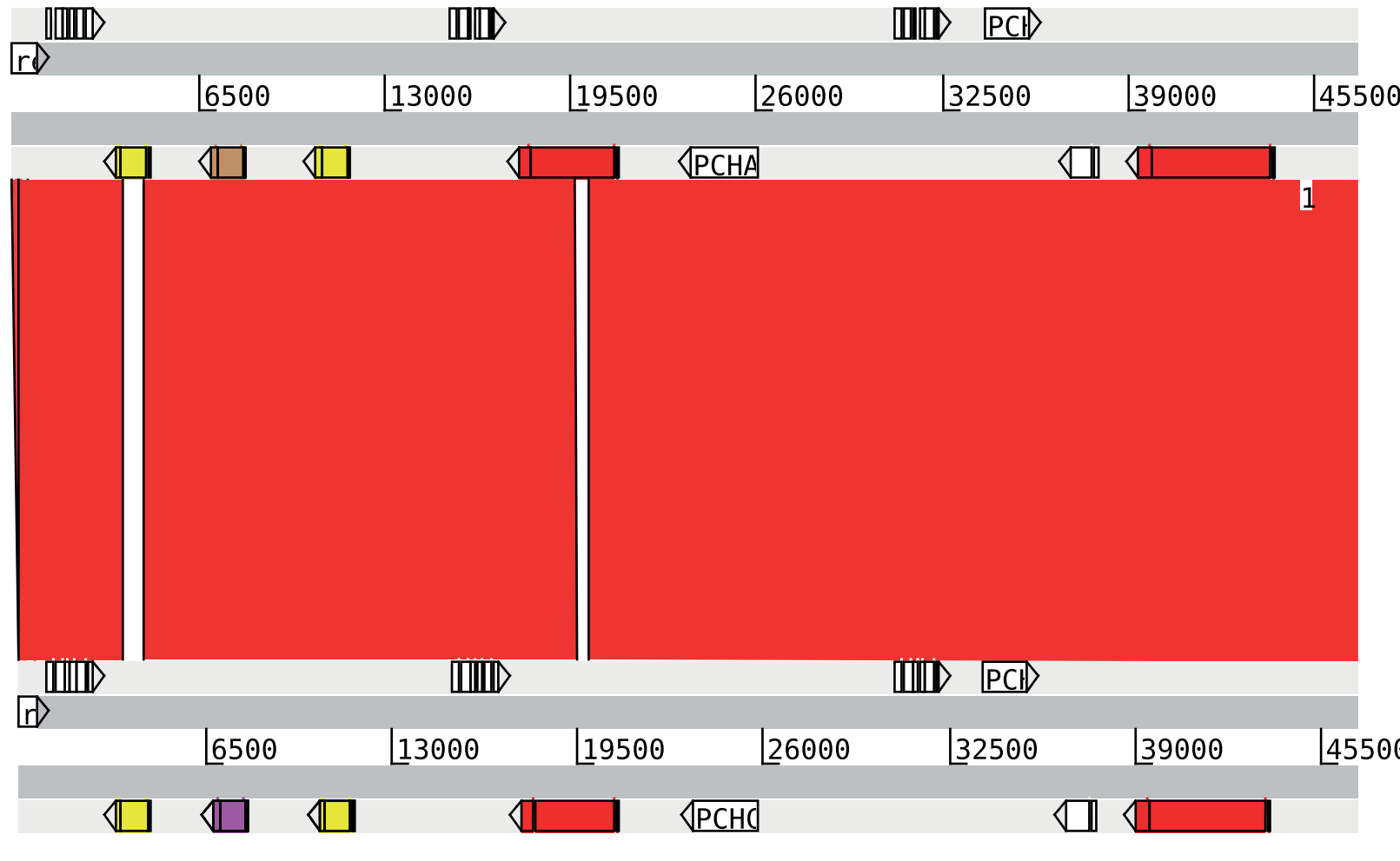

11L

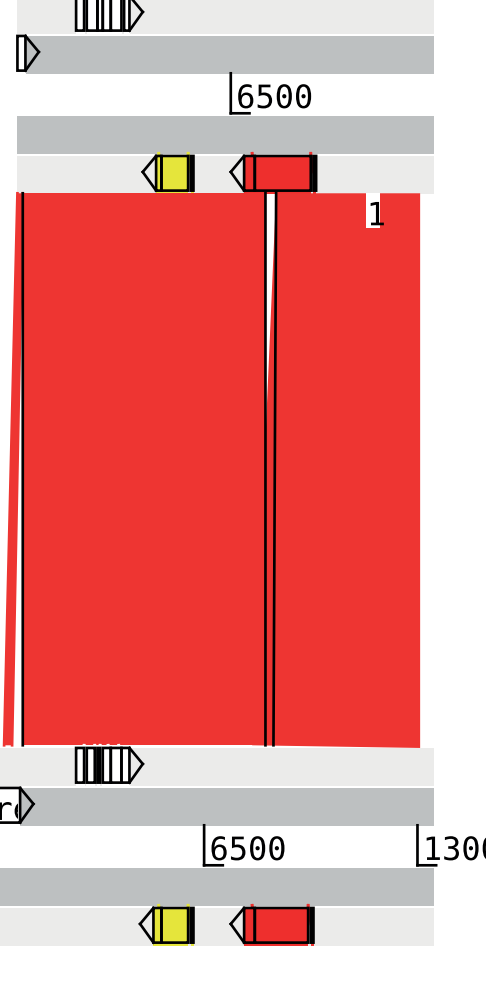

12L

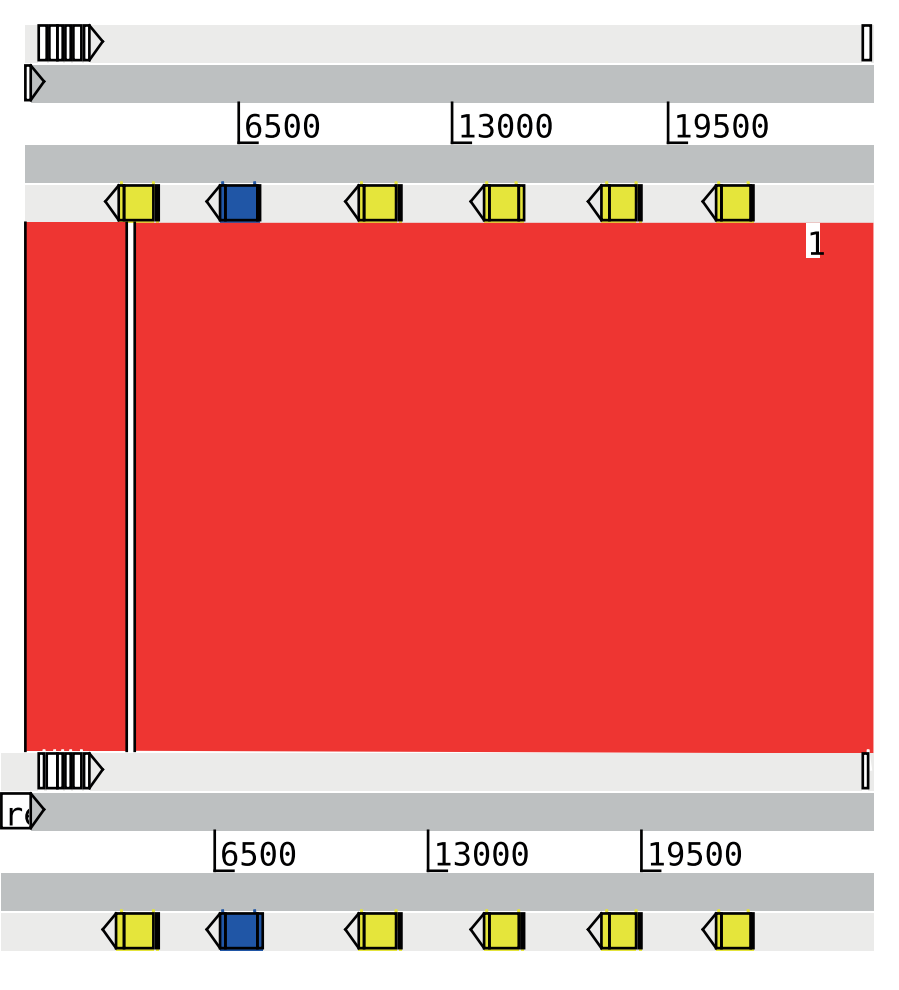

13L

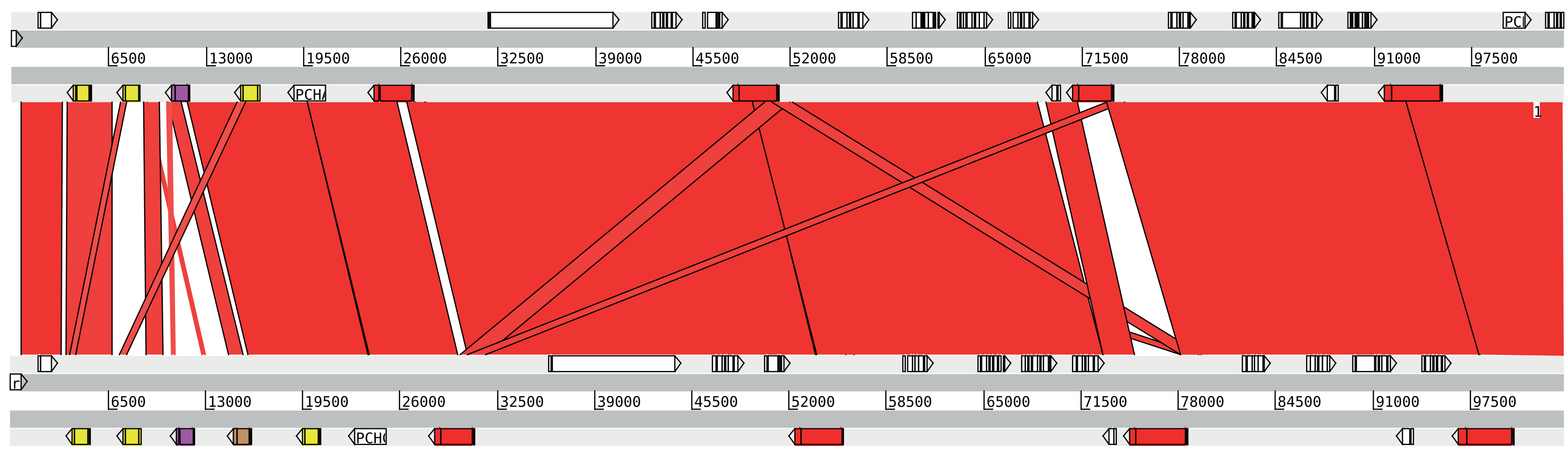

14L

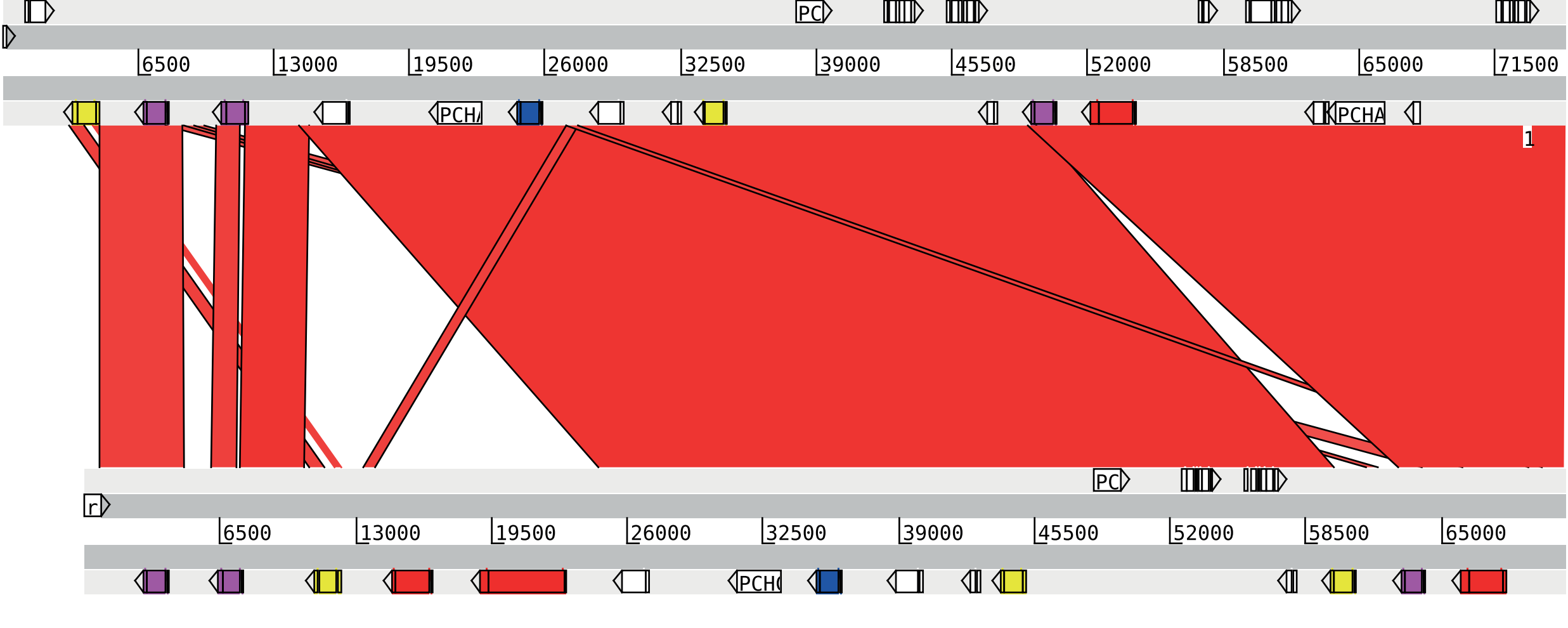

1R

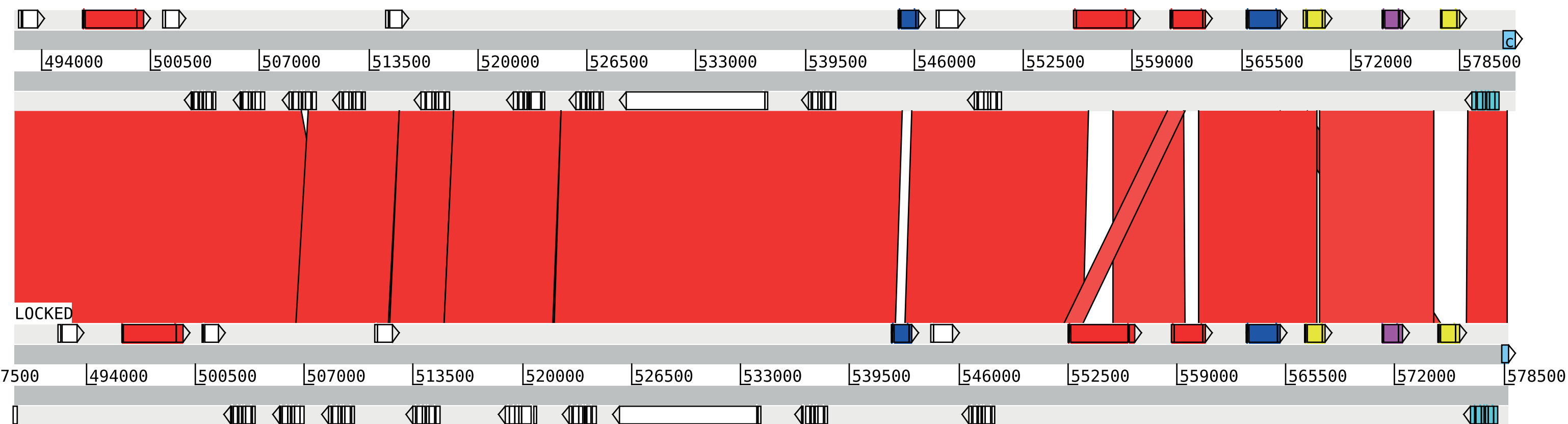

2R

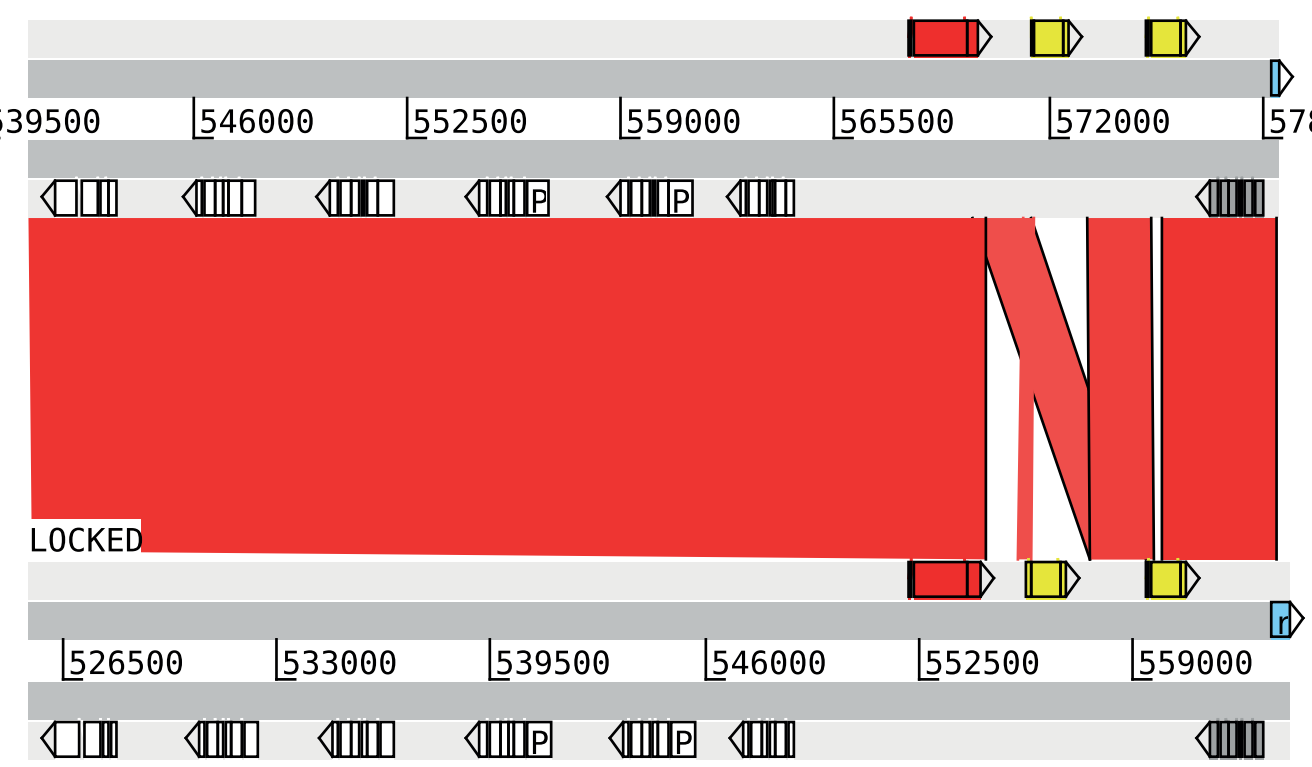

3R

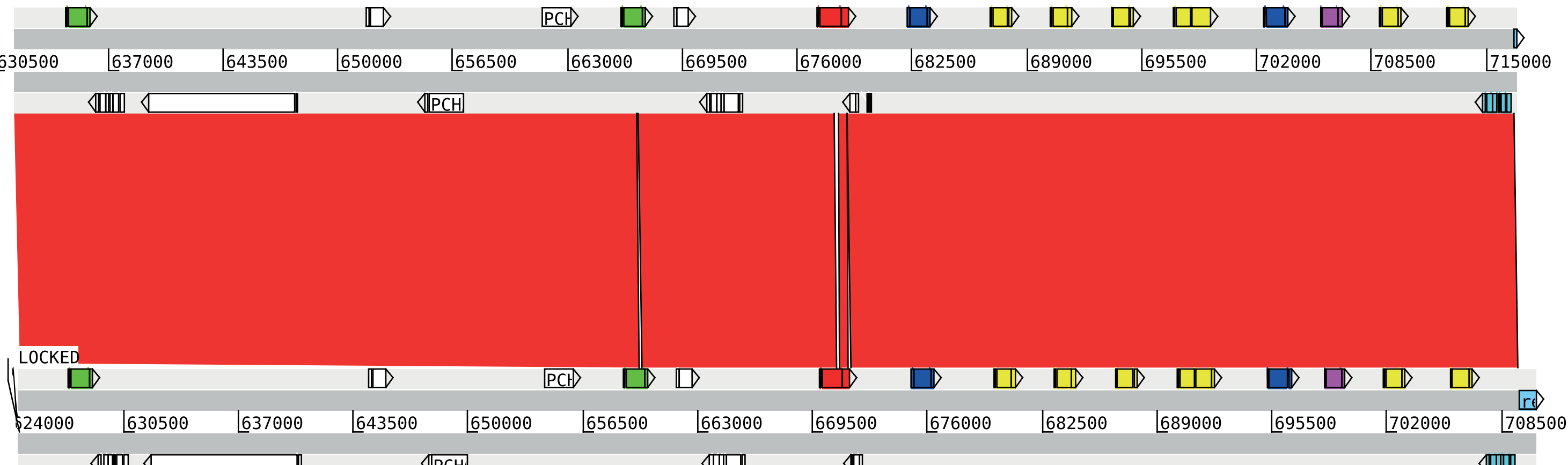

4R

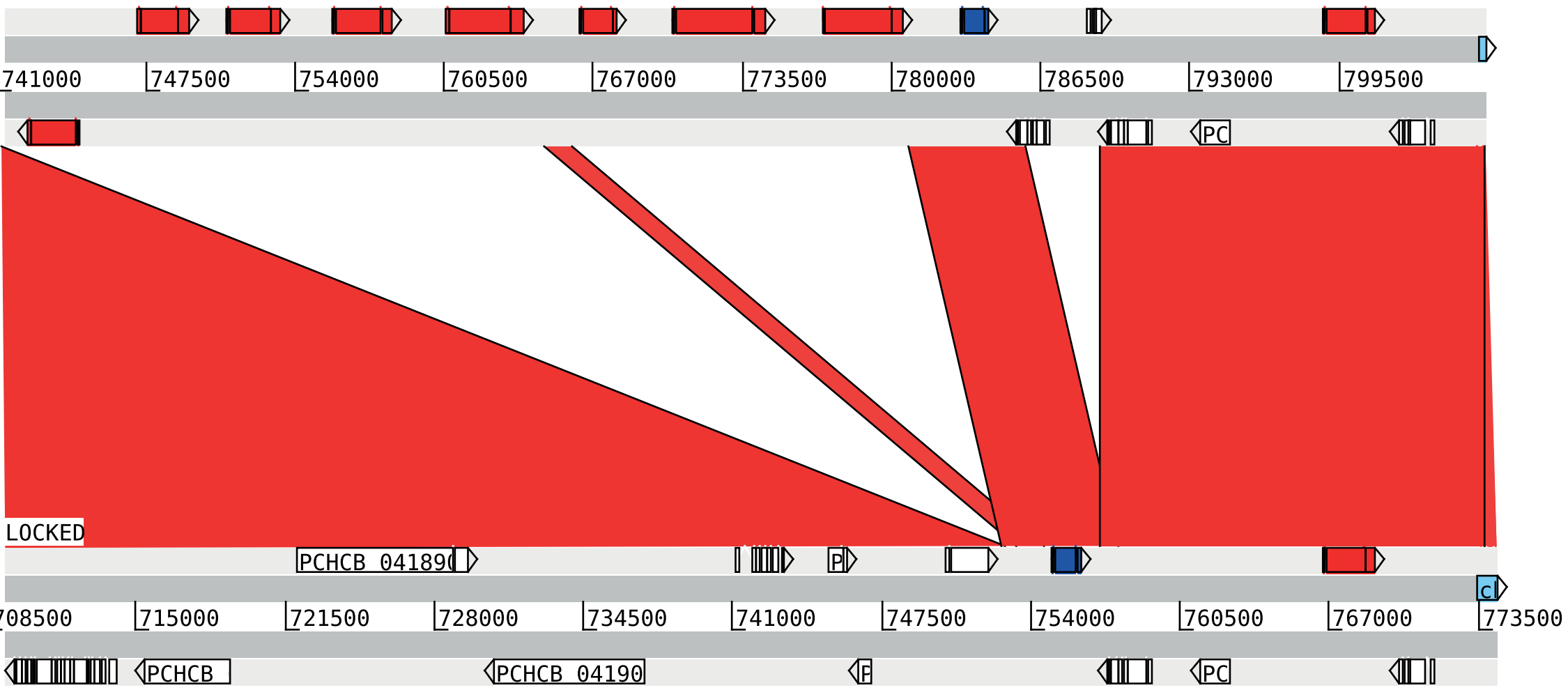

5R

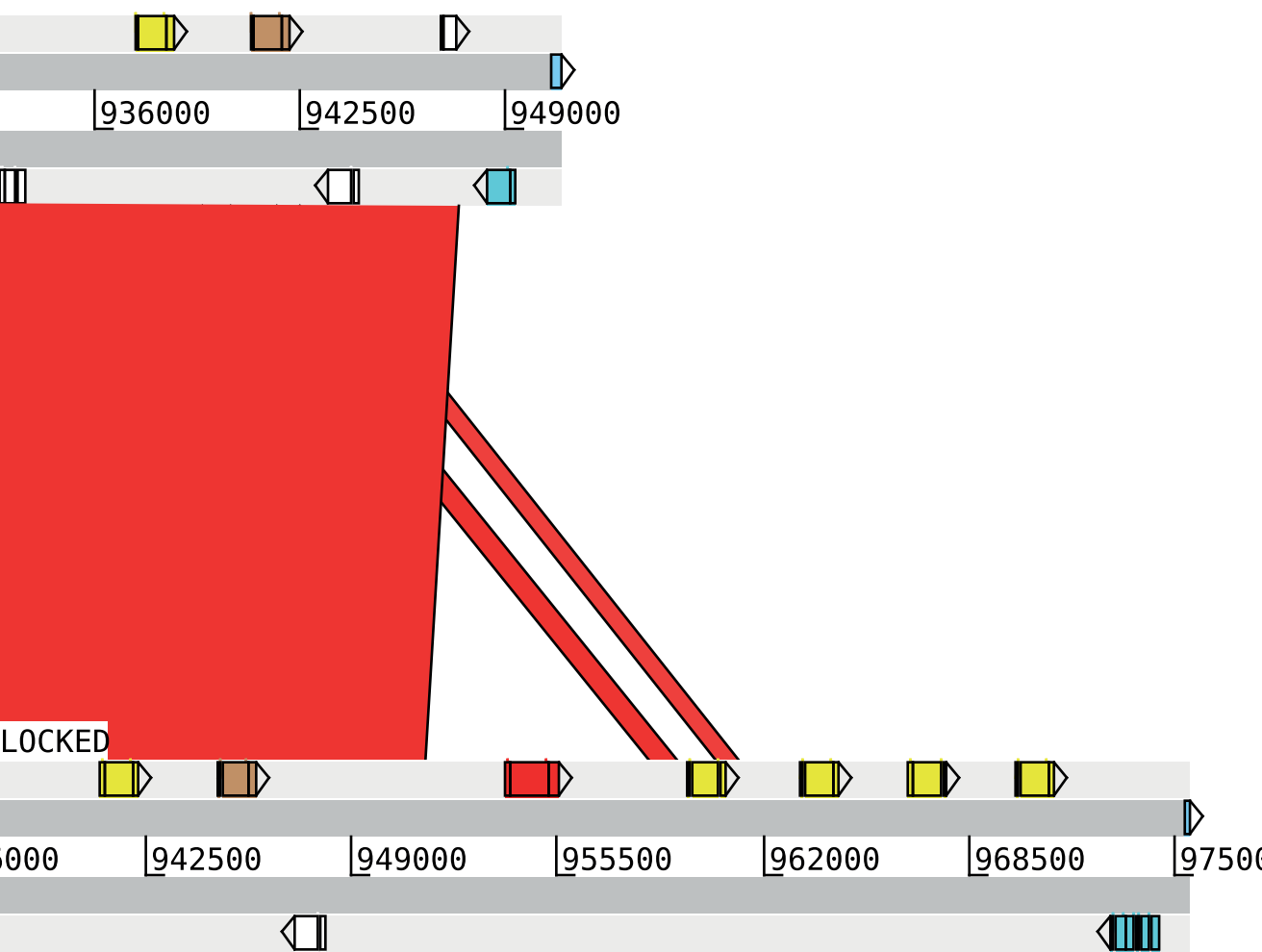

6R

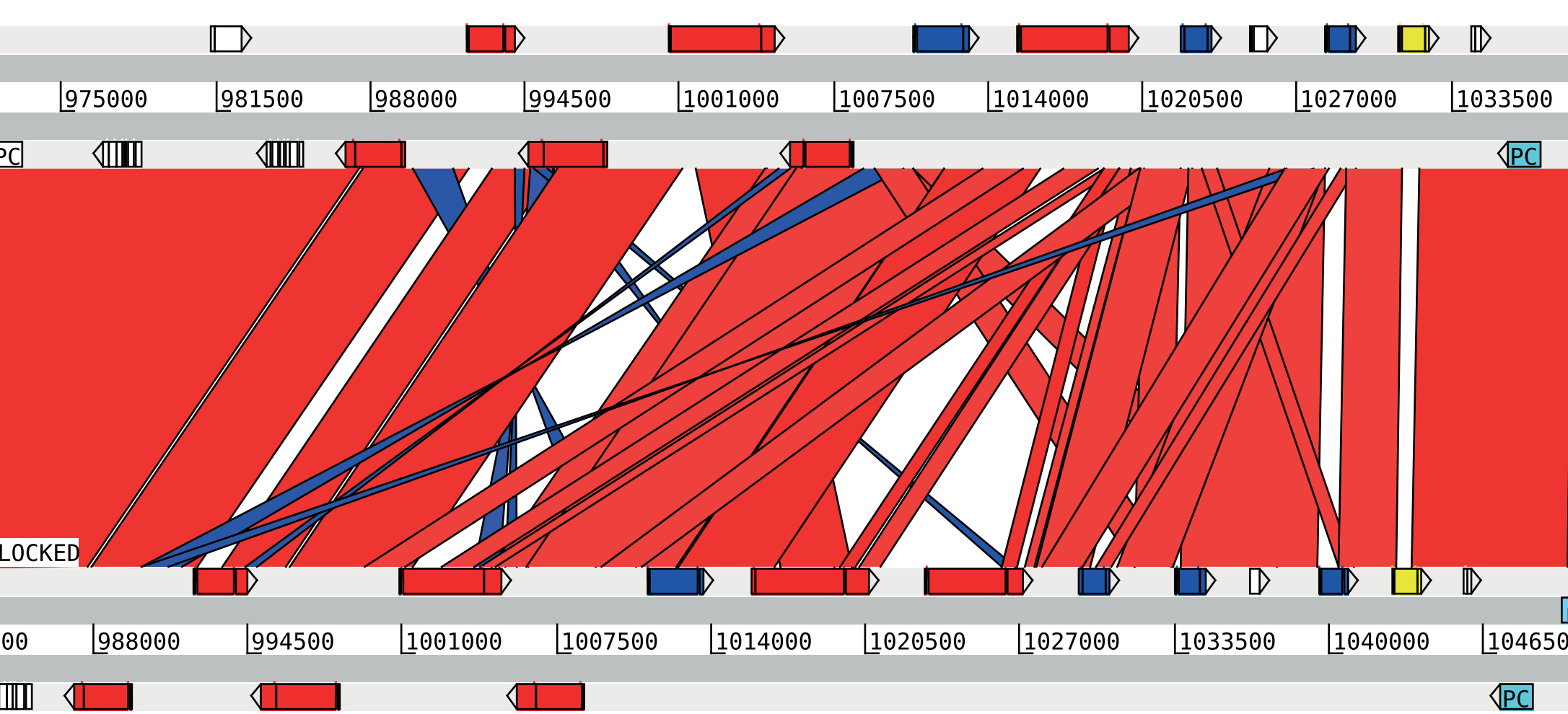

7R

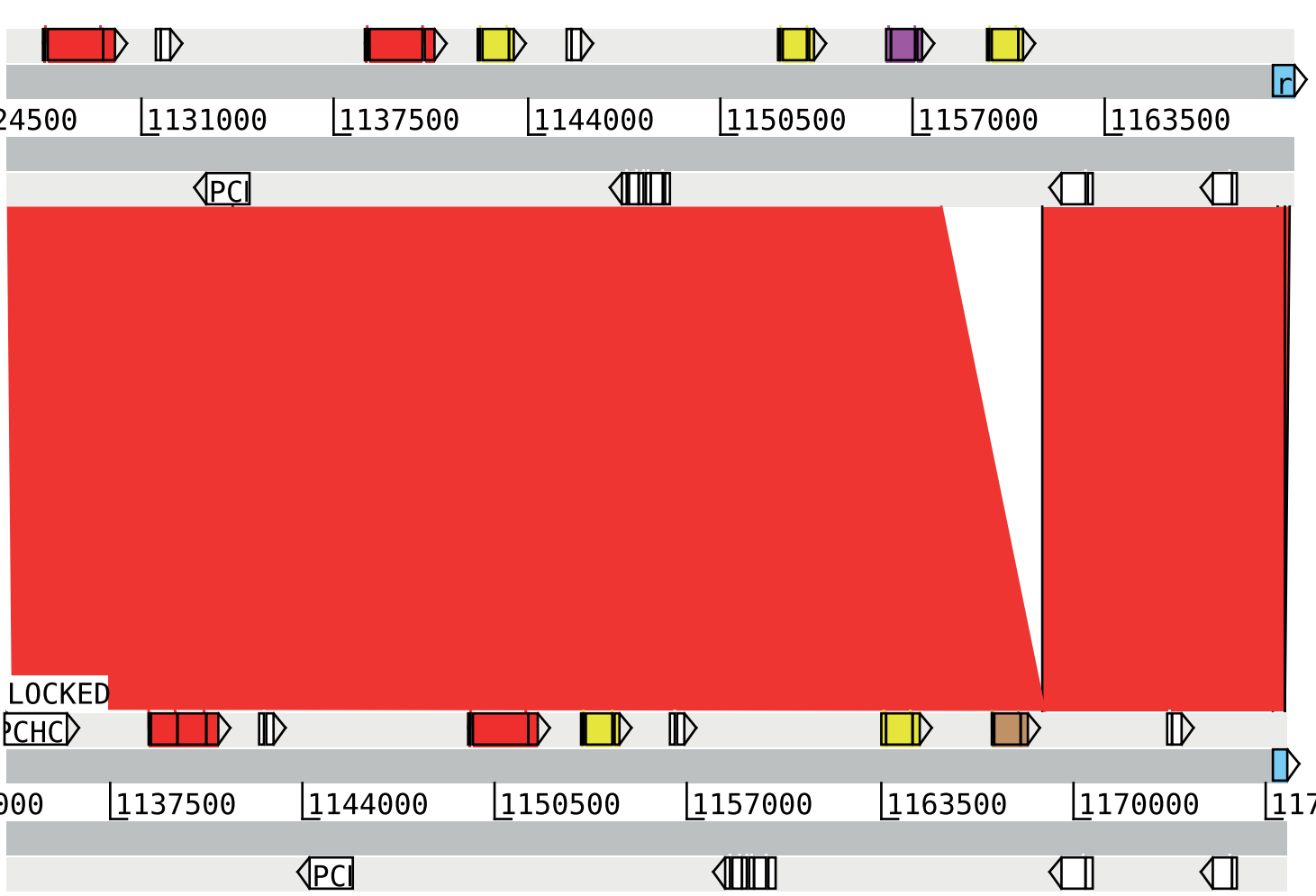

8R

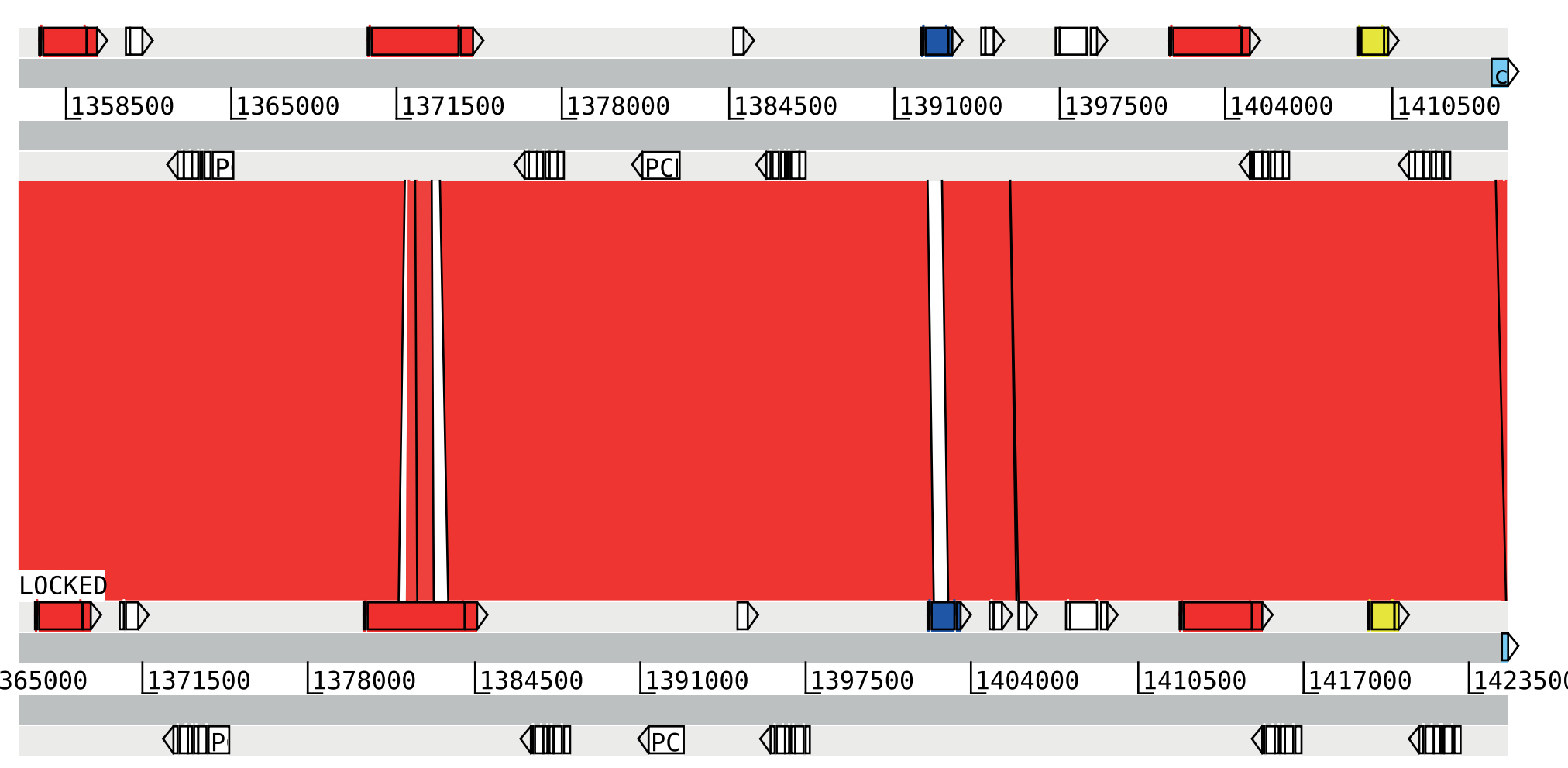

9R

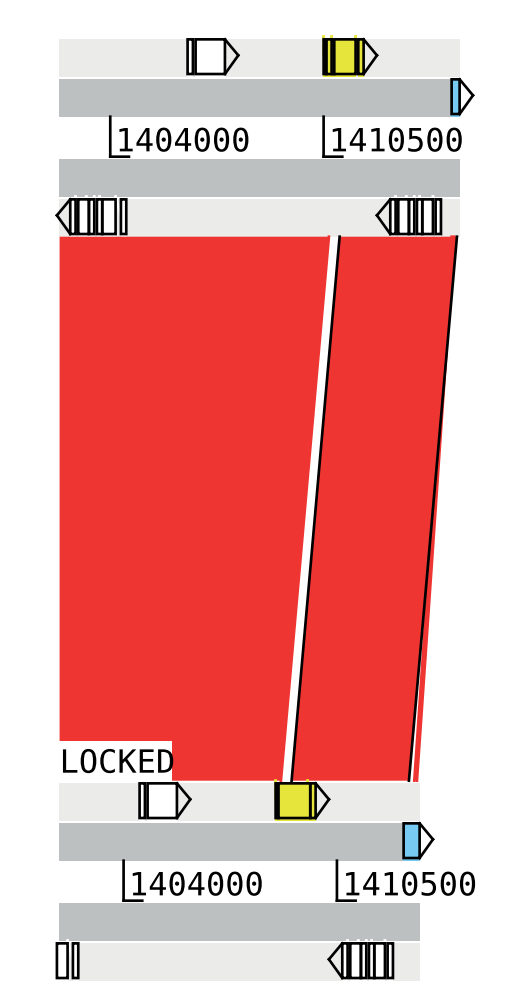

10R

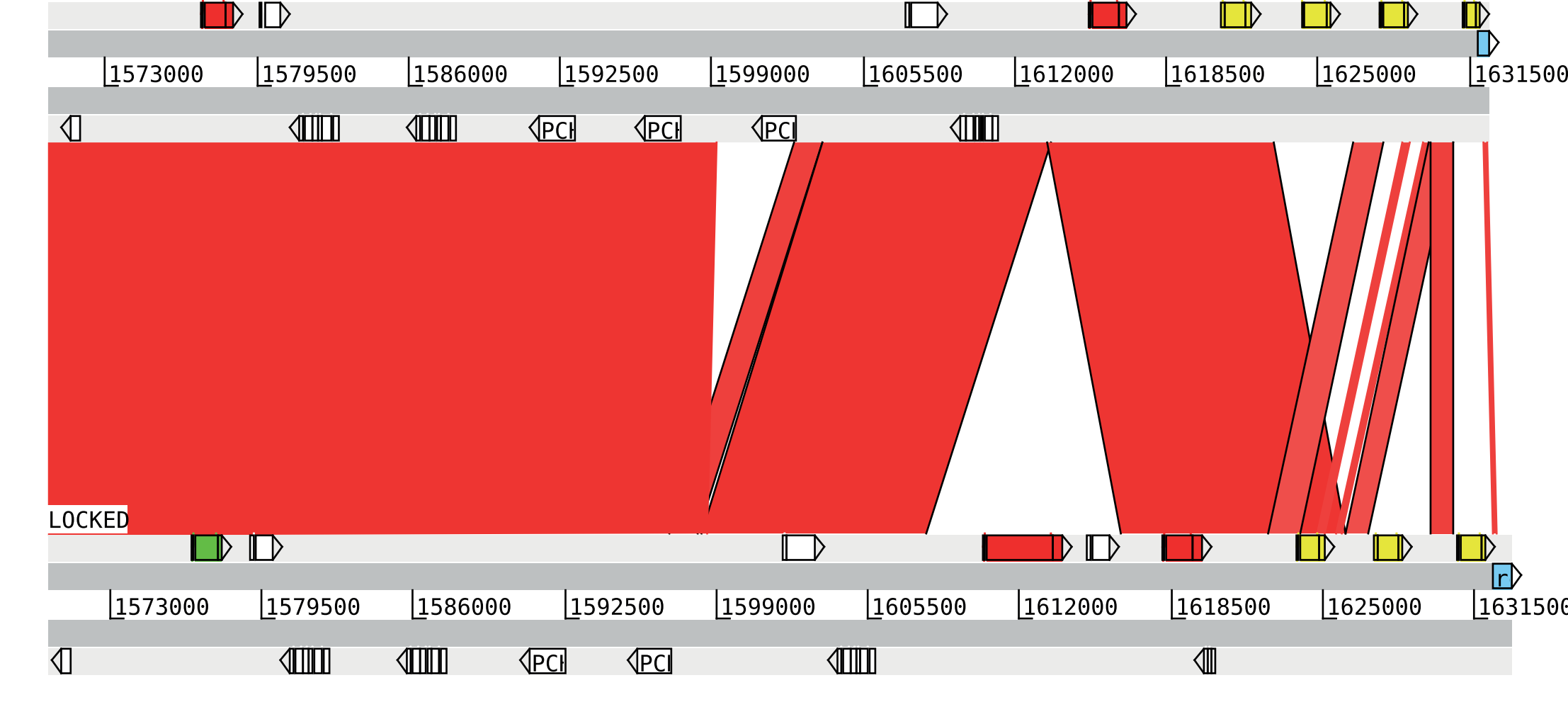

11R

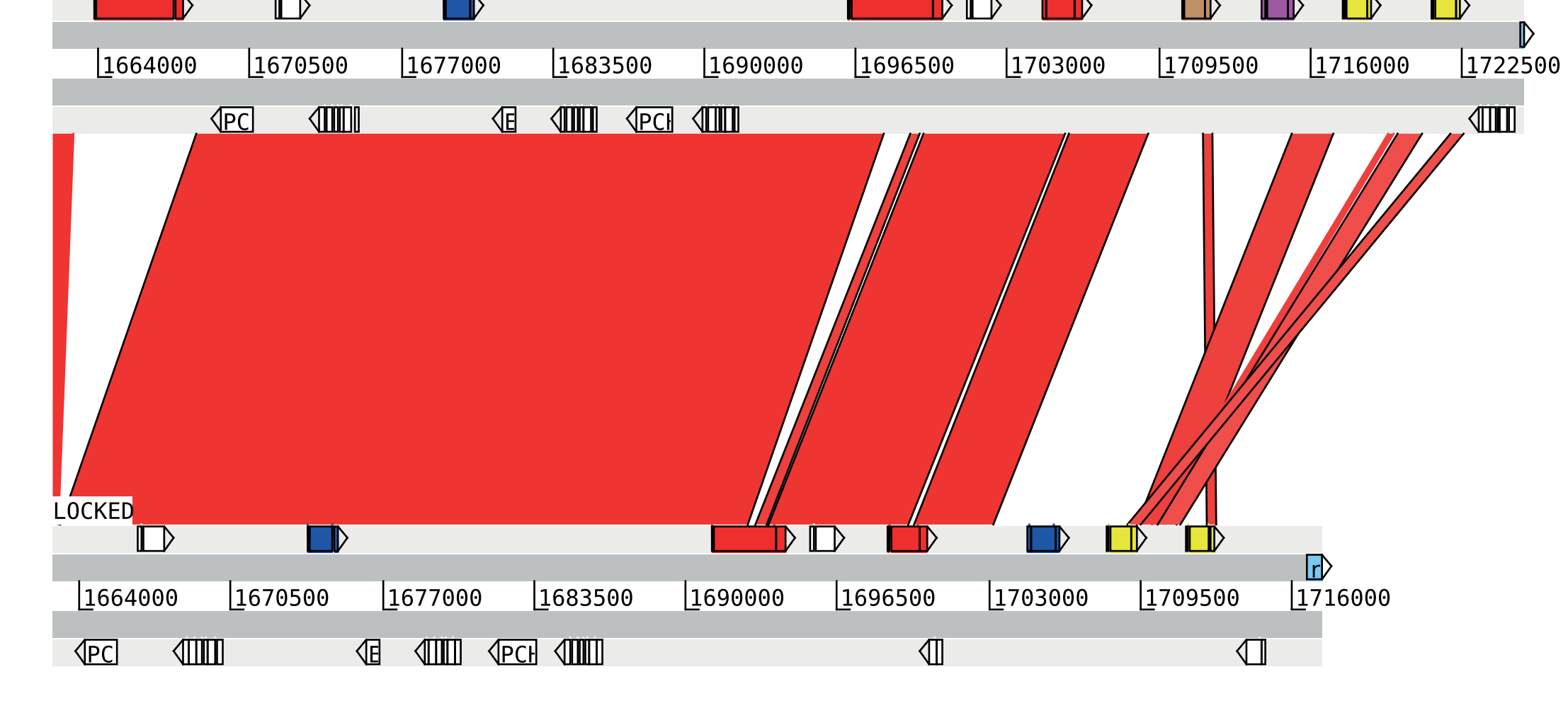

12R

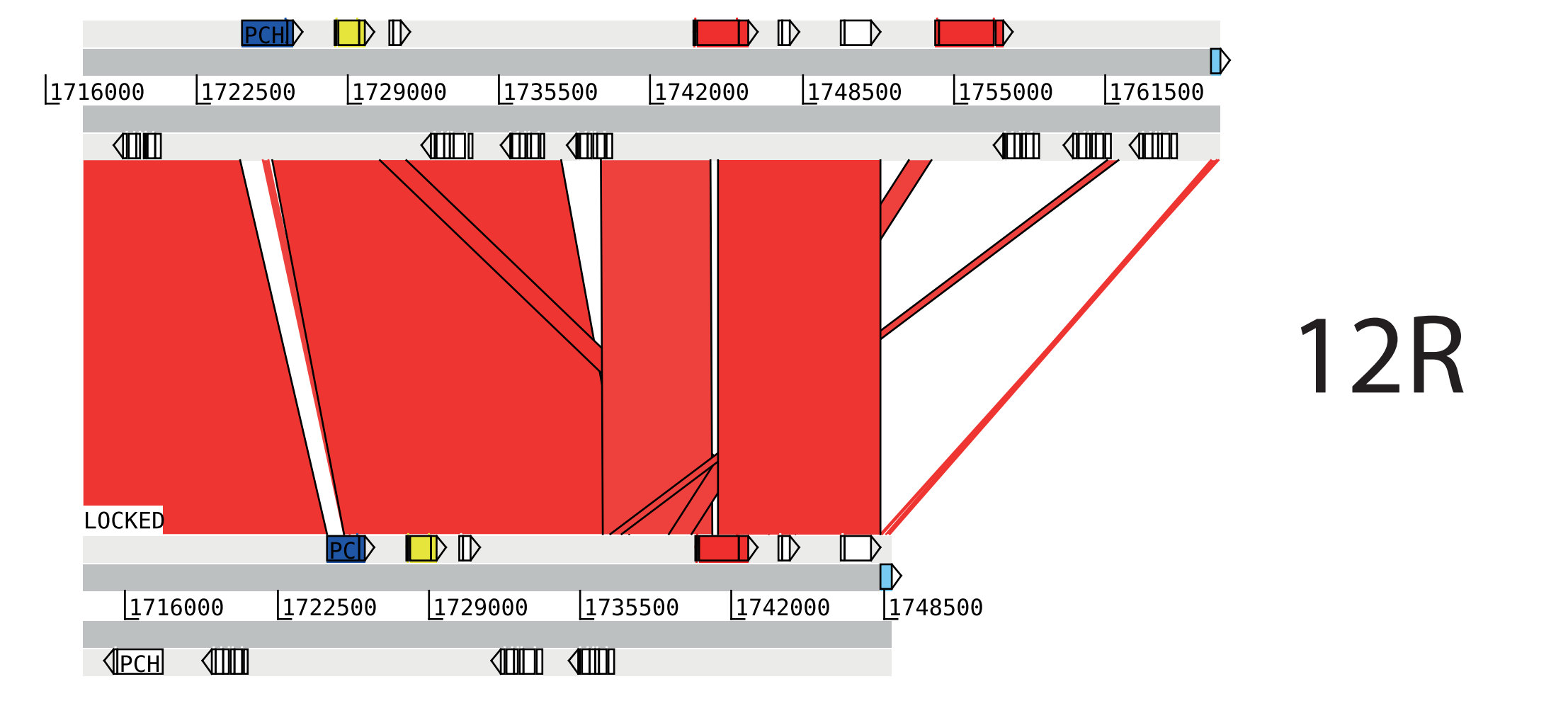

13R

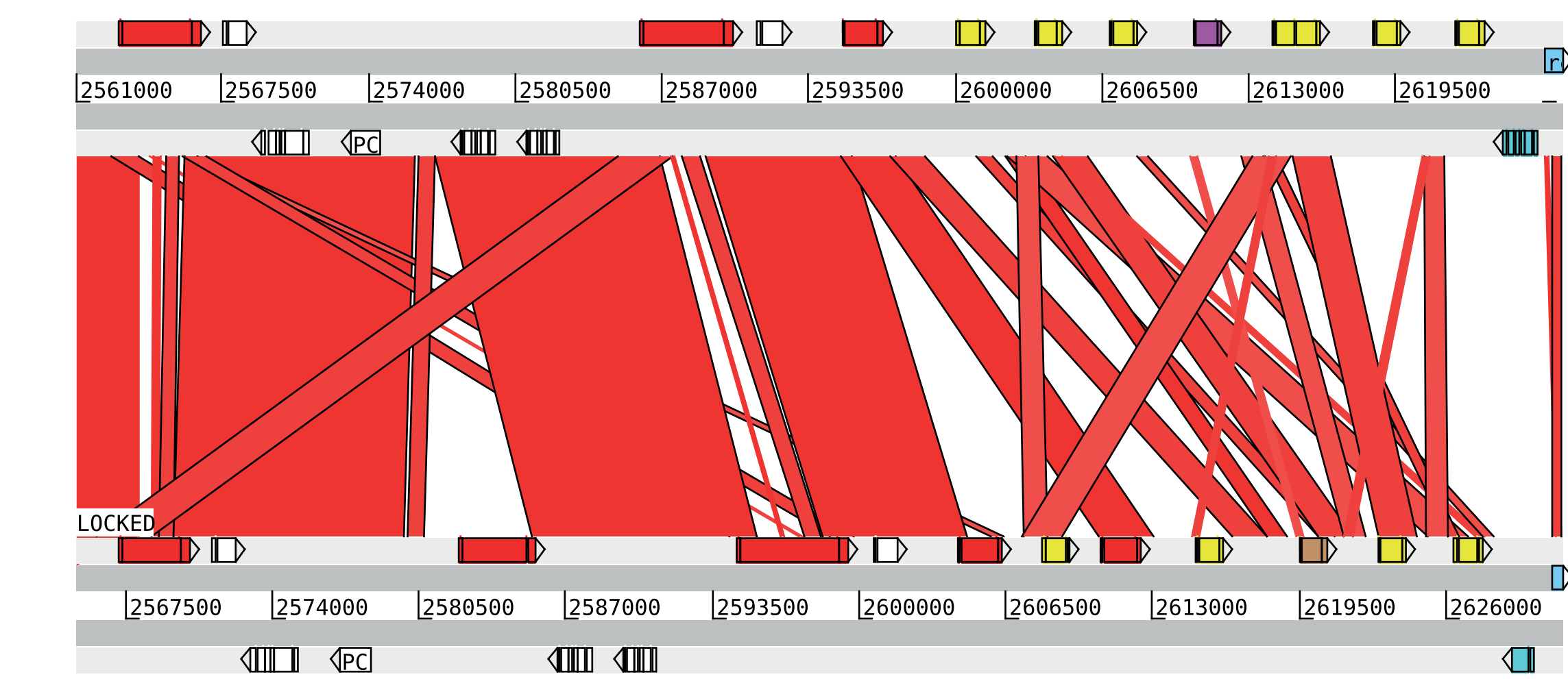

14R

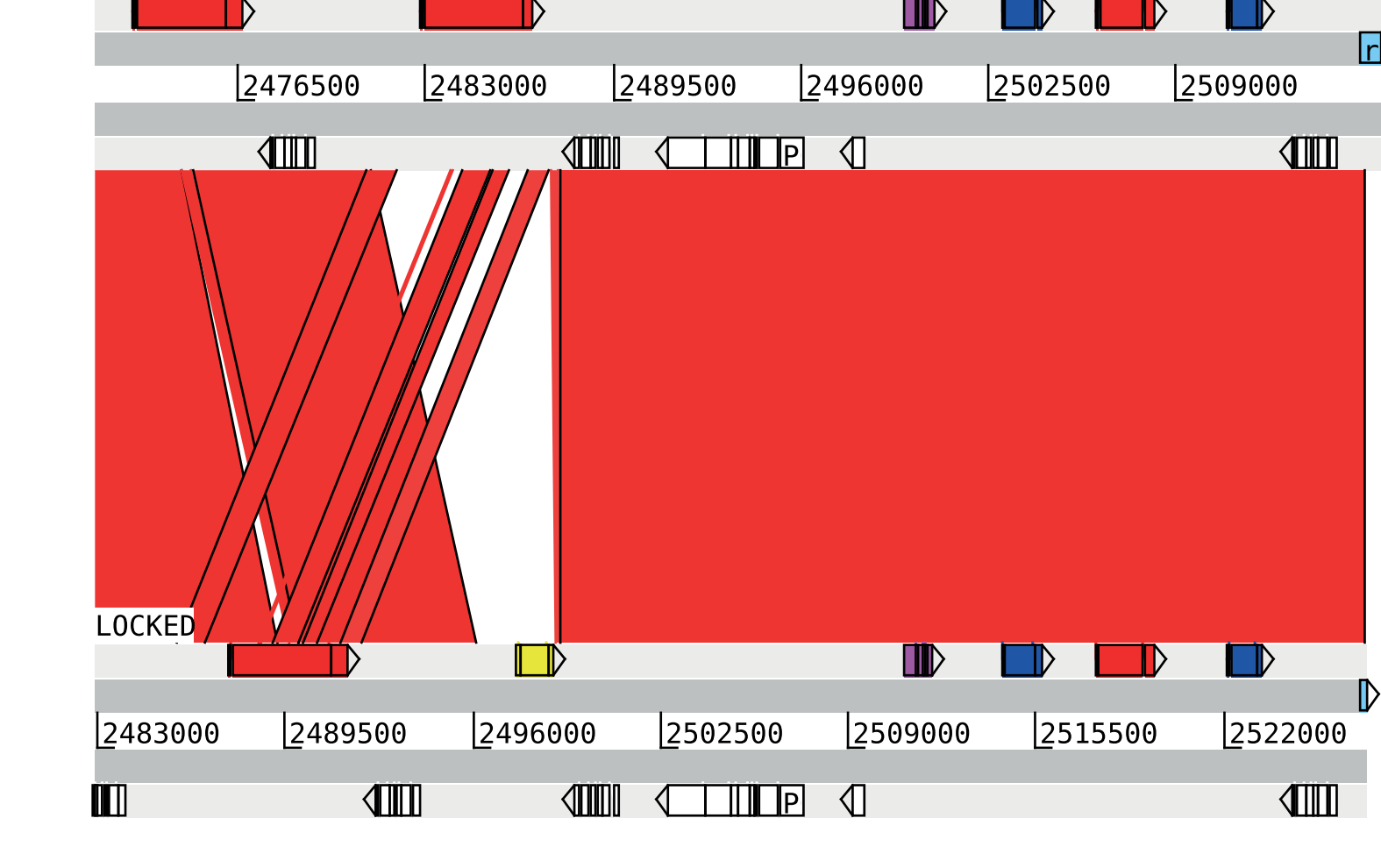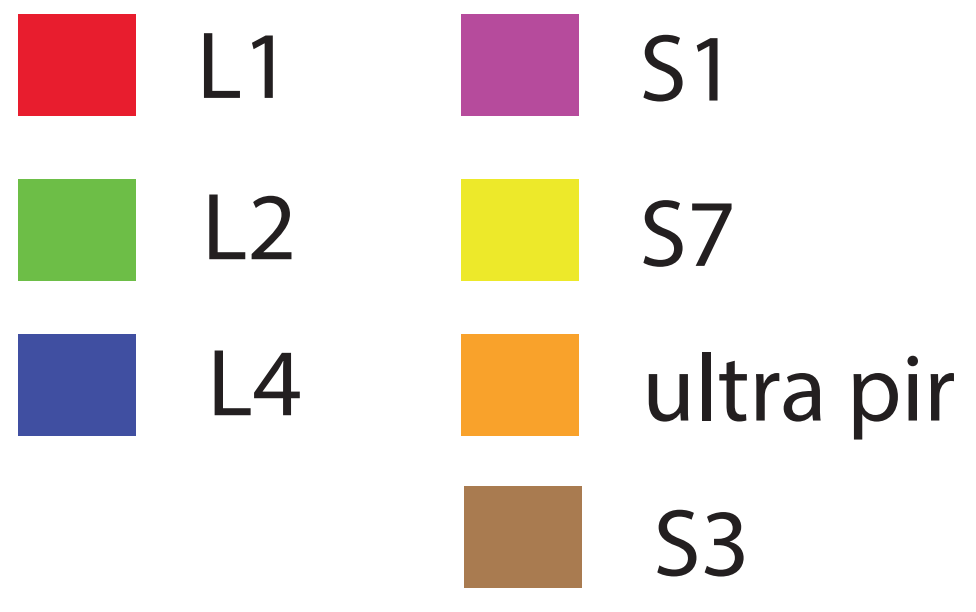

Supplement: Supplementary file 1 [file wellcomeopenres-3-16303-s0000.tgz › 62ddac39-263a-43ae-b82b-c9c68471a205_Supplementary_Figure_1.pdf]

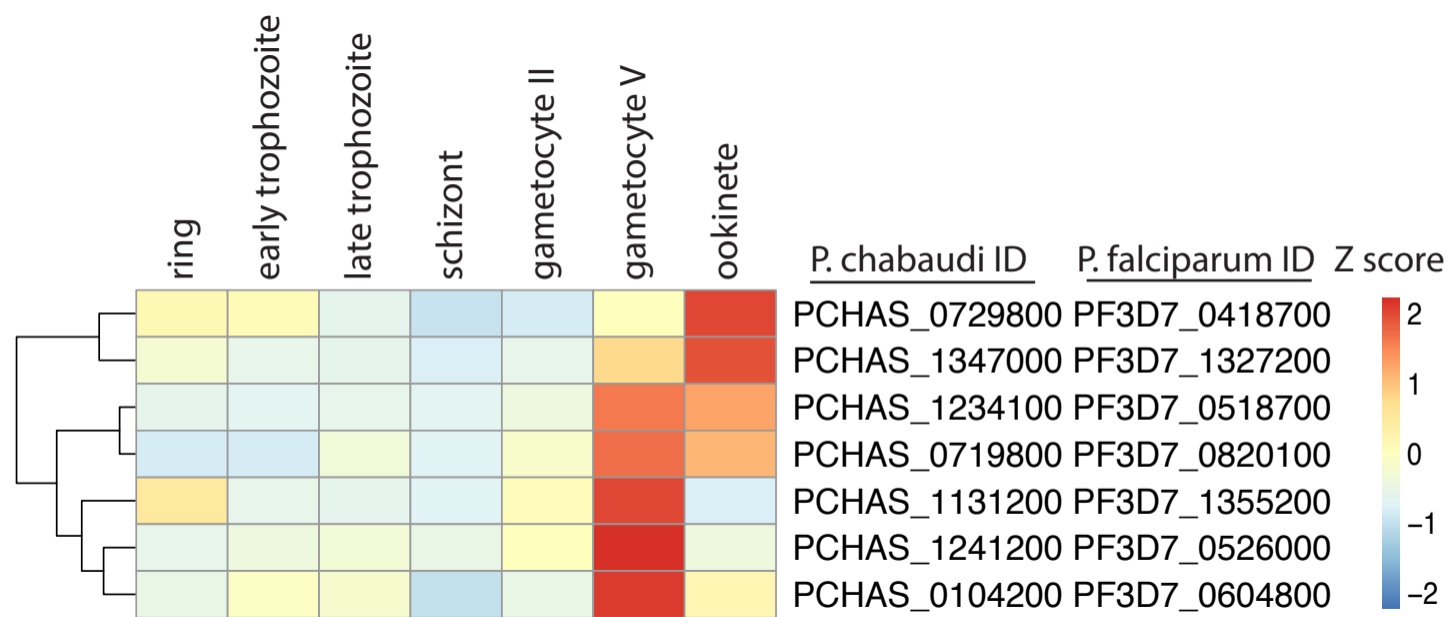

Supplement: Supplementary file 2 [file wellcomeopenres-3-16303-s0001.tgz › d664566b-e31a-46e8-92d9-55812134cb8b_Supplementary_Figure_2.pdf]
