# Supplementary material for: Genomic and transcriptomic comparisons of closely related malaria parasites differing in virulence and sequestration pattern
Source: Wellcome Open Res. 2018 Dec 6;3:142. Originally published 2018 Nov 2. [Version 2] doi: 10.12688/wellcomeopenres.14797.2 (PMC6259598; doi:10.12688/wellcomeopenres.14797.2)
Supplement: Peer review history

# Peer review: Referee response for version 1

**DOI:** 10.21956/wellcomeopenres.16125.r34198

**Published:** 2018-11-26

**Authors:** Matthew L. Aardema

**Affiliations:** 1 Sackler Institute for Comparative Genomics, American Museum of Natural History, New York City, NY, USA; 2 Department of Biology, Montclair State University, Montclair, NJ, USA

Overall, I found the study this manuscript describes to be well-thought out and thorough, and the paper itself exceptionally well written. Its analysis of divergent genes and patterns of gene expression between two phenotypically distinct strains of *P. chabaudi *is an important step towards helping the malaria research community discover the causes of variation in virulence among human malaria species. In particular, this work strongly suggests continued analysis of the pir genes in P. falciparum may prove fruitful for better understanding and prediction of patterns of infection. For this reason, I recommend this manuscript be accepted for indexing in Wellcome Open Research.

 My one and only suggestion is that too often it felt like results were being needlessly described in earlier sections of the manuscript. For example, the second to last paragraph of the introduction could and perhaps should be removed.

 Two minor points: First, please clarify whether the *pir* reference genes used in *pir* gene identification were also protein sequences, or rather nucleotide sequences. This is not clear in the text.

 Second, in the abstract, two consecutive sentences start with ‘however’. I would suggest changing one of them.

I have read this submission. I believe that I have an appropriate level of expertise to confirm that it is of an acceptable scientific standard.

# Response

**Published:** 2018-11-26

**Authors:** Adam Reid

**Affiliations:** Wellcome Trust Sanger Institute, UK

Thank you very much for taking the time to review our manuscript. Thanks also for your suggestions for improvement. We agree that the second to last paragraph of the introduction was superfluous and have removed it. We have also removed a ‘however’ from the abstract.

*Two minor points: First, please clarify whether the pir reference genes used in pir gene identification were also protein sequences, or rather nucleotide sequences. This is not clear in the text.*

 Protein sequences were used in both cases. This has been clarified in the text.

# Peer review: Referee response for version 1

**DOI:** 10.21956/wellcomeopenres.16125.r34183

**Published:** 2018-11-20

**Authors:** Xin-Zhuan Su

**Affiliations:** 1 Malaria Functional Genomics Section, Laboratory of Malaria and Vector Research, National Institute of Allergy and Infectious Diseases (NIAID), National Institutes of Health (NIH), Bethesda, MD, USA

This manuscript describes genomic and transcriptomic comparisons of two Plasmodium chabaudi strains (PcAS and PcCB), focusing on the distribution and expression of some subtelomeric genes such as the Pir and Fam-a/b/c genes. Growth characteristics and invasion phenotypes of the two parasites were also investigated and compared. The assembling of the PcCB genome into 14 chromosomes with 0 gap is impressive, which provides an important resource for future studies. The comparison of Yir gene expression between the parasites derived from mosquito infection and blood stage passage is also interesting; however, follow-up experiments are required in order to identify individual genes playing a role in virulence. Finally, genes under diversifying selection were identified, most of which were those involved in parasite-host interaction such as invasion of red blood cells. It would be nice to also present data on genome-wide polymorphisms (SNPs, indels, and CNVs), which will give readers a general picture of genetic differences between the two parasites. I cannot comment on technical details of genome analysis methods. I do trust the Sanger genomic team for good quality of analyses.

 This is a well analyzed and written manuscript. I only have a few minor comments:

 1, Page 11, Figure 4 legend: Subfigure A, B, and C are not properly identified; please consider adding a subtitle for each. The (A) and (C) for AAPL and ChAPL in the legend can be misleading for subfigures.

 2, Page 11, left column, line 6 from the bottom: Remove ‘and’ to read “…invasion are under…’.

 3, Page 11, right column, line 5: ‘is’ should be ‘in’? 

 4, Table 2. Consider adding a row of summary numbers.

 5, Supplementary files: Excel format with a title and explanations for the terms in each table will make it easier to read. I understand that one can open the text files in Excel, but it would be nice to be able to read and understand the numbers in the same screen.

I have read this submission. I believe that I have an appropriate level of expertise to confirm that it is of an acceptable scientific standard.

# Response

**Published:** 2018-11-26

**Authors:** Adam Reid

**Affiliations:** Wellcome Trust Sanger Institute, UK

Thank you very much for taking the time to review our manuscript. We have acted on most of your suggestions and adjusted the text accordingly. A couple of other issues we discuss below.

*It would be nice to also present data on genome-wide polymorphisms (SNPs, indels, and CNVs), which will give readers a general picture of genetic differences between the two parasites.*

We feel that we have addressed SNPs in the form of the dN/dS analysis and CNVs within genes in the orthologue analysis. Indels are more difficult to interpret as they do not follow a well-understood evolutionary model. We felt that they would not provide additional useful information regarding the evolution of virulence in these organisms at this time

5, Supplementary files: Excel format with a title and explanations for the terms in each table will make it easier to read. I understand that one can open the text files in Excel, but it would be nice to be able to read and understand the numbers in the same screen.

We agree that this would be more useful, but we were asked to submit plain .csv files.
